# Slip Buddy App for Weight Management: Randomized Feasibility Trial of a Dietary Lapse Tracking App

**DOI:** 10.2196/24249

**Published:** 2021-04-01

**Authors:** Sherry Pagoto, Bengisu Tulu, Molly E Waring, Jared Goetz, Jessica Bibeau, Joseph Divito, Laurie Groshon, Matthew Schroeder

**Affiliations:** 1 University of Connecticut Department of Allied Health Sciences Storrs, CT United States; 2 Worcester Polytechnic University Foisie Business School Worcester, MA United States

**Keywords:** mobile app, mHealth, weight loss, obesity, diet, mobile phone

## Abstract

**Background:**

Although calorie tracking is one of the strongest predictors of weight loss in behavioral weight loss interventions, low rates of adherence are common.

**Objective:**

This study aims to examine the feasibility and acceptability of using the Slip Buddy app during a 12-week web-based weight loss program.

**Methods:**

We conducted a randomized pilot trial to evaluate the feasibility and acceptability of using the Slip Buddy app compared with a popular commercial calorie tracking app during a counselor-led, web-based behavioral weight loss intervention. Adults who were overweight or obese were recruited on the web and randomized into a 12-week web-based weight loss intervention that included either the Slip Buddy app or a commercial calorie tracking app. Feasibility outcomes included retention, app use, usability, slips reported, and contextual factors reported at slips. Acceptability outcomes included ratings of how helpful, tedious, taxing, time consuming, and burdensome using the assigned app was. We described weight change from baseline to 12 weeks in both groups as an exploratory outcome. Participants using the Slip Buddy app provided feedback on how to improve it during the postintervention focus groups.

**Results:**

A total of 75% (48/64) of the participants were female and, on average, 39.8 (SD 11.0) years old with a mean BMI of 34.2 (SD 4.9) kg/m2. Retention was high in both conditions, with 97% (31/32) retained in the Slip Buddy condition and 94% (30/32) retained in the calorie tracking condition. On average, participants used the Slip Buddy app on 53.8% (SD 31.3%) of days, which was not significantly different from those using the calorie tracking app (mean 57.5%, SD 28.4% of days), and participants who recorded slips (30/32, 94%) logged on average 17.9 (SD 14.4) slips in 12 weeks. The most common slips occurred during snack times (220/538, 40.9%). Slips most often occurred at home (297/538, 55.2%), while working (153/538, 28.4%), while socializing (130/538, 24.2%), or during screen time (123/538, 22.9%). The conditions did not differ in participants’ ratings of how their assigned app was tedious, taxing, or time consuming (all values of *P*>.05), but the calorie tracking condition gave their app higher helpfulness and usability ratings (all values of *P*<.05). Technical issues were the most common type of negative feedback, whereas simplicity was the most common type of positive feedback. Weight losses of ≥5% of baseline weight were achieved by 31% (10/32) of Slip Buddy participants and 34% (11/32) of calorie tracking participants.

**Conclusions:**

Self-monitoring of dietary lapses and the contextual factors associated with them may be an alternative for people who do not prefer calorie tracking. Future research should examine patient characteristics associated with adherence to different forms of dietary self-monitoring.

**Trial Registration:**

ClinicalTrials.gov NCT02615171; https://clinicaltrials.gov/ct2/show/NCT02615171

## Introduction

### Background

Obesity is a major risk factor for type 2 diabetes [1], but many people still do not have access to evidence-based lifestyle interventions [2]. Technology-delivered lifestyle interventions may have increased reach, and early studies reveal promising impacts, but they are still fairly burdensome and expensive [3]. A key source of burden is self-monitoring in the form of calorie tracking, which requires a person to record all of the food and beverage they consume each day. Although adherence to calorie tracking predicts weight loss outcomes [4], adherence is notoriously low [5]. A recent study found that rates of consistent calorie tracking in a web-based weight loss program fell from 68% in week 1 to 21% by week 12 [6]. Interventions that do not use calorie tracking have failed to produce weight loss outcomes [7]. Thus, new forms of self-monitoring that can produce similar or greater weight loss are needed.

Interestingly, calorie tracking is a relatively complex form of self-monitoring compared with forms used for other behaviors. For example, in smoking cessation interventions, the smoker keeps a tally of the number of cigarettes smoked each day and notes the triggers associated with each smoking episode [8]. Similarly, a simpler way to perform dietary self-monitoring could be to have people track only the segment of their diet that requires intervention, that is, the eating episodes that account for excess calories or dietary lapses. Dietary lapses are considered *nonhomeostatic eating*, which includes any eating episode that occurs in excess of one’s needs to maintain health, not just in terms of food quantity but also food quality [9]. To the extent that we can help people identify and eliminate dietary lapses, we may be able to affect energy balance without the task of calorie tracking.

Previous ecological momentary assessment studies have revealed that people are able to identify and self-monitor dietary lapses [10-13]. Carels et al [12] had 12 dieters track their dietary lapses over the course of a week along with contextual factors surrounding the lapses. The results showed that hunger, location, negative affect, and certain activities such as watching television and socializing were common during lapses. Stress, hunger, and socializing were found to be predictors of dietary lapses in other studies [13,14]. This research has stimulated at least two dietary lapse tracking apps designed for use in behavioral weight loss interventions [15,16]. OnTrack allows users to track their dietary lapses from the WW (formerly Weight Watchers) points-based weight loss program and the contextual factors surrounding those lapses. The app sends 6 prompts per day asking the users to record 17 contextual factors (eg, mood, hunger, and temptations), the data from which go into a machine learning algorithm that drives just-in-time intervention messages that occur when the user is at high risk for lapses [10,15]. In a randomized trial, adding OnTrack to WW’s points-based weight loss program was found superior in weight loss outcomes over 10 weeks compared with the WW program alone, but it did not appear to enhance weight loss when added to a version of the WW program that was less intensive in terms of point tracking [15]. In contrast to OnTrack, which requires users to perform WW point tracking, the Slip Buddy app was designed as a replacement for traditional forms of dietary tracking.

Employing a user-centered design, we developed the Slip Buddy app, which allows users to track dietary lapses as they occur and the contextual factors that cued each lapse [16]. To best mimic the way calorie tracking apps are used, users are only expected to record at the time of recordable eating episodes. However, unlike traditional calorie tracking apps that require the user to record all intake every day, the burden of using Slip Buddy declines as dieters become more skilled at avoiding lapses simply because the only task of the app is to track lapses. Slip Buddy works such that users simply hit an *Oops!* button each time they experience a dietary lapse, which are referred to as slips in the app based on feedback regarding preferred language from the target population during the design process. When a user logs a slip, the app asks them to rate stress and degree of hunger to satiety on scales of 0 to 10 and to name the location (eg, home and work) and activity (eg, watching television and socializing) they were engaging in at the time of the slip and the food consumed. The app passively collects day and time information and maintains a log of all slips. A tab called *Slip History* shows the user’s entire history of slips, including contextual data (eg, stress rating and location) so that users can learn about the circumstances that trigger their slips. Each morning, the user receives a notification to complete a daily check-in that involves recording how long they slept, their stress and hunger level, and their weight that morning if they stepped on the scale. Each afternoon, they receive a notification to complete check-in on their stress and hunger levels. At the end of the day, users receive a notification asking if they recorded all of their slips for the day and, if not, to record any remaining slips. In a usability study, we evaluated the use and acceptability of Slip Buddy in 16 adults over 4 weeks. Participants used the app 26.8 out of 28 days, tracked about 14 slips, and lost on average 1.5% (SD 0.7%) of their baseline weight, even though no other intervention was provided. In that study, we then used Slip Buddy data to generate predictive models that informed intervention messages to display to the user when they were in a situation in which overeating was triggered in the past. Participants used Slip Buddy for another month while receiving these messages, but participant feedback revealed that they did not find the messages more helpful than slip tracking alone, so we dropped that aspect of the app. As Slip Buddy was not designed as a standalone weight loss intervention, but instead as an alternative to calorie tracking tools used in behavioral weight loss interventions, the next step is to examine the feasibility and acceptability of Slip Buddy as a replacement for the traditional calorie tracking app in a web-based behavioral weight loss intervention (Trials Registration: Clinicaltrials.gov NCT02615171).

### Objectives

This study is a pilot feasibility randomized controlled trial in which participants with overweight or obesity were randomized to receive either the Slip Buddy app or a commercial calorie tracking app during a 12-week counselor-led web-based weight loss intervention. Our first aim is to compare groups on retention and app use to explore whether the commercial app is significantly superior to Slip Buddy, which would point to the need for further modification to Slip Buddy before proceeding to a fully powered efficacy trial. Our second aim is to describe the total number of slips reported and the contextual factors reported at slips, including the location of slips, type of eating episode (eg, lunch and snack), stress, and hunger/satiety. This aim is descriptive in nature. Our third aim is to assess the usability, acceptability, and burden of the Slip Buddy app quantitatively and via qualitative interviews where participants shared what they liked and disliked about the app and the features they would like added. Similar to aim 1, we tested whether the commercial app was significantly superior in terms of usability, acceptability, and burden, which would signal areas for further modification to Slip Buddy before proceeding to an efficacy trial. Our fourth aim is to describe the percentage of weight loss from baseline to 12 weeks in both groups, including the proportion of participants who lost clinically significant weight. This aim is exploratory because only a fully powered efficacy trial could address this question.

## Methods

### Study Design, Settings, and Participants

We conducted a pilot feasibility randomized trial in which participants who were overweight or obese were recruited into a remotely delivered intervention via web-based advertisements at the University of Connecticut, ResearchMatch, and Facebook groups across the United States between July and October 2019. All work was approved by the University of Connecticut Institutional Review Board. We recruited people interested in losing weight with BMI between 27 and 45 kg/m^2^, aged 18-65 years, who had an Android smartphone, and who had phone connectivity at home and work. Exclusion criteria were inability to walk unaided for one-fourth mile without stopping, not being a daily Facebook user (because the group-based part of the intervention was delivered via Facebook), taking medications known to affect appetite and/or weight, having a condition that precludes dietary changes (eg, ulcerative colitis), type 1 or 2 diabetes, gastric bypass surgery or plans to do so during the study period, pregnancy or lactation, severe mental illness or substance use disorder, binge eating disorder, or loss of 5% or more weight in the past 3 months. Recruitment ads contained a link to a screening survey that included a study description, initial informed consent, and screening questions. Participants eligible after the screening survey were emailed the consent form and completed a telephone screening call. The screening call included reviewing the consent form, any remaining eligibility-related questions, and an emailed link to the baseline survey.

Before randomization, potential participants were required to attend an orientation webinar in which study staff used a methods-motivational interviewing approach to help them understand the scientific rationale of the trial design, research questions, and methods. This helps participants understand the commitment entailed in trial enrollment and helps set clear expectations (eg, transparency about the length of assessments), explain the scientific rationale for procedures (eg, randomization and feasibility versus efficacy testing), diffuse ambivalence about research participation using motivational interviewing techniques, and make explicit commitments to self and trial methods. Upon completion, those interested in proceeding with the study were mailed a Wi-Fi scale (Fitbit Aria) and asked to provide staff with log-in information for the scale so that weight could be recorded for assessments. After randomization, participants had a 60-minute call with a study staff person to receive guidance on how to download and use their assigned app and enter their assigned Facebook group. Participants were allowed to keep the scale and were compensated for completing the assessments.

### Intervention Conditions

Participants in both conditions were assigned one of the diet tracking apps and received the Diabetes Prevention Program (DPP) lifestyle intervention delivered within a counselor-led private Facebook group that included all participants in their respective conditions. Participants randomized to the Slip Buddy condition were provided the Slip Buddy app, and participants randomized to the calorie tracking condition were instructed to install the free, commercially available MyFitnessPal app. Each group had a different counselor who was either a registered dietitian or a clinical psychologist, and each was trained in the app assigned to their respective conditions and led the Facebook group for that condition.

As in our previous work [17,18], the lifestyle intervention was delivered via twice daily posts, and each week’s content was based on the corresponding module of the DPP. The DPP assigns participants the goals of (1) calorie tracking to achieve a calorie goal based on the amount needed to lose 1-2 lb (0.45-0.90 kg) per week (modified to weekly slip tracking in the Slip Buddy condition), (2) developing a healthy diet consistent with the American Heart Association guidelines, (3) engaging in 150 to 300 minutes per week of moderate-intensity exercise, (4) developing a strength training regimen consistent with the National Guidelines for Physical Activity, and (5) losing 1-2 lb per week. All DPP content related to dietary self-monitoring was modified in the Slip Buddy condition to focus on slip tracking (as opposed to calorie tracking). Goal setting occurred on Monday mornings when the counselor asked participants to set 2 to 3 diet and exercise goals and gave specific suggestions based on the topic of the week (eg, self-monitoring, reducing added sugar, and adding 15 minutes of exercise) to help participants progress toward their weight loss goal. On Fridays, the counselor put up a weigh-in post asking participants to reply with their weight change in pounds (eg, lost 1 pound) for the week. This ensures participants weigh themselves at least once a week on the study scale and allows an opportunity for problem solving for those who do not lose weight. This procedure is in place of private weigh-ins that typically occur between the counselor and each participant before group meetings in clinic-based programs. Conducting the weigh-ins as a discussion thread requires less time than having the counselor do individual weigh-ins for each participant, which is a procedure that does not scale well in groups that are as large as 32 participants. Goal accountability occurred every Sunday when the counselor asked participants to report how they did on their weekly goals. In between these key posts were posts related to the topic of the week (eg, nutrition and making time for exercise). On the remaining days, the counselor posted discussion threads relating to that week’s module. Each week, staff produced weight and engagement reports for the counselors so they could identify participants who had not engaged in the past week and/or who were not losing weight, and they attempted to engage them in the group by tagging them in posts. Tagging a participant in a post results in them receiving a notification on their Facebook account that, when clicked, leads them to the post in which they are tagged. In our previous studies, this Facebook-delivered weight loss intervention produced mean weight losses at 12 weeks ranging from 2.6% to 4.8% [18,19].

#### Slip Buddy App

**Figure 1:**
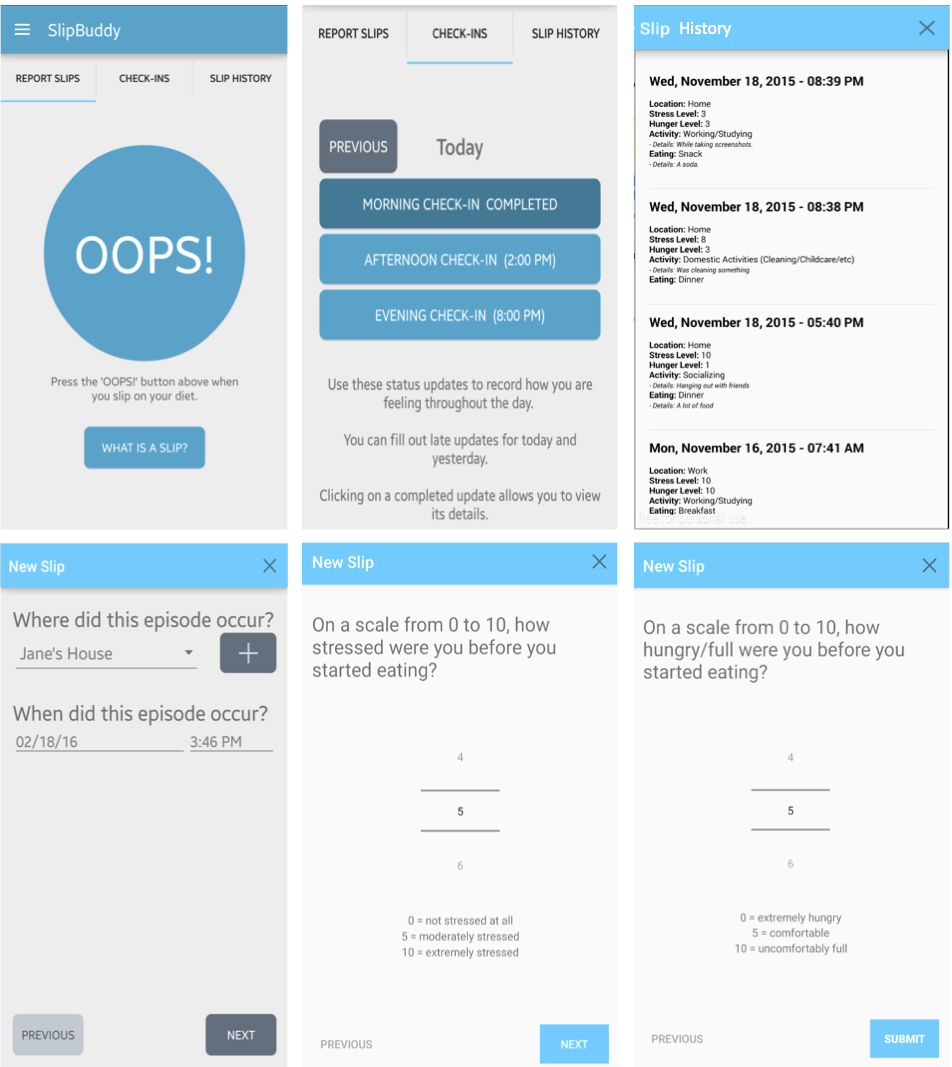
Slip Buddy Screen Shots.

As described above, we developed the Slip Buddy app, which assists users in tracking nonhomeostatic eating and the contextual factors surrounding it (Figure 1) [16]. As *nonhomeostatic eating* is scientific jargon, the app refers to these episodes as *slips*. Participants were instructed to hit an *Oops!* button each time they had a diet slip, defined as any eating that resulted in consuming (food or drink) more than planned at a meal or between meals, eating in the absence of hunger (eg, ate a donut someone brought to work), emotional eating, eating past the point of fullness, or an unhealthy food choice (eg, stopped for fast food instead of cooking). The definition of a slip appeared near the *Oops!* button as a reminder to the user. To increase awareness of when slips are most likely to occur, the app passively collected the date and time for each slip reported. For each slip, participants were asked to rate their stress and degree of hunger to satiety on a 0 to 10 scale to describe the context of the slip from drop-down menus, including the type of eating episode (eg, lunch and snack) and activity during the episode (eg, working, socializing, and watching television). They were also asked to type in their location (eg, restaurant), food consumed, and any other notable details they wanted to remember later in open text boxes. A check-in tab asks participants to report weight, hours slept last night, stress and hunger in the morning, and stress and hunger in the afternoon, but unlike our first pilot study, we removed the notifications for the morning and afternoon check-ins to keep notifications to a minimum. The only notification occurred at the end of the day, asking participants if they missed entering any slips for the day and, if so, to record the missed slips. The data collected by the app were securely sent to the remote Slip Buddy database server in addition to being recorded in the local database on participants’ mobile phones. The history tab showed participants’ past slip entries so they could look for patterns in contextual factors such as stress ratings, hunger level, activities, and/or location (Figure 1). Just as the calorie tracking group was given guidance on how to learn from calorie tracking, each week, the counselor instructed Slip Buddy participants to view their slip history from the previous week and use that information to set goals around how to avoid and/or manage cues associated with past slips. For example, if most slips occurred while watching television in the evening, they could set the goals of planning healthy snacks at this time or reducing television time. Participants were urged to use the app to learn when and why they slip and to reduce their slips over time toward the goal of losing 1-2 lb per week. In the Facebook group, the DPP content related to calorie tracking and cues was modified to address slip tracking and to draw participants’ attention to the eating cues they were learning about from their Slip Buddy history. No additional content or other modifications were made to the DPP content in the Slip Buddy condition.

#### Calorie Tracking Condition

Participants randomized to the calorie tracking condition were instructed to download MyFitnessPal, a free, commercially available mobile app that provides users with a personalized calorie goal and allows them to track their caloric intake and energy expenditure via exercise in an effort to stay within that goal. Participants were asked to enter everything they eat and drink throughout each day and all of their structured physical activity. They were asked to stay within their calorie goals to facilitate a weight loss of 1-2 lb per week. The group counselor instructed participants to use MyFitnessPal daily and to inspect their dietary entries for high-calorie foods that could be eliminated to achieve their calorie goal.

### Measures

#### Retention

Retention was defined as the percentage of participants in each condition who completed the 12-week follow-up measures, which included weigh-in and a survey.

#### App Use

We report 3 metrics of app use: (1) whether participants used their assigned app at least once during the 12-week intervention, (2) the total number and percentage of possible days participants used their app over the 12 weeks, and (3) whether participants used their assigned app at least once in week 12 (ie, sustained engagement). As the nature of diet tracking differed between the 2 treatment conditions, how we assessed app use also differed. For participants in the Slip Buddy condition, we intended to categorize participants as having used the Slip Buddy app for a given day if (1) backend data from the app revealed at least one slip was recorded or check-in completed (optional) or (2) in the absence of slips, the participants responded to the end-of-day check-in saying that they did not have any slips that day. Staff reviewed the data in the Slip Buddy database server (ie, backend data) and recorded the number of days each week each participant used the app (eg, either recorded a slip or responded to a notification or check-in reporting that they experienced no slips). However, some participants reported that they did not see or receive the end-of-day check-in notifications from the app, which we determined was related to the authentication token on the phone expiring periodically. The end-of-the-day notification gives the participant the opportunity to confirm that no slips occurred if none had been recorded thus far. Without the notification, we could not distinguish between a day in which the participant did not track slips and a day in which no slips occurred. For this reason, backend data would be an underestimation of app use. As failure can sometimes occur while transmitting app data to the remote database server, we also collected self-report app use data by emailing participants each week a single item asking them how many days they used the app to track slips that week. Self-report data were available for 74.4% (282/379) of weeks across all participants (counting only 7 weeks for the participant who withdrew because of pregnancy). As 26% of self-reported data were missing and backend data were incomplete by an unknown amount, we leveraged both forms of data to measure app use. We used the larger of the 2 values for 2 reasons: (1) when self-reported data are higher than backend data, it could correct for the underestimation bias of backend data and (2) when backend data are higher than self-report, it could correct for recall bias from self-report. The weakness is that we do not have a way to correct for recall bias from self-report that overestimates use, which surely exists to some extent. Self-report data were used for 58.1% (220/379 weeks) of the total weeks, and backend data were used for 27.4% (104/379 weeks) of weeks, which includes the 7 weeks when the backend data were higher than the self-report data and the 97 weeks in which self-report data were missing. On the remaining 14.5% (55/379 weeks) of weeks, self-report and backend data were the same so that the value was used. Although there is no way to correct for possible overestimations via self-report, on 25.6% (97/379 weeks) of weeks, only backend data were available, which would be an underestimate for those weeks. As a sensitivity analysis, we calculated app use using self-reported data if available, and when self-report data were not available, backend data were used. These metrics only differ from the main analysis for the 7 weeks (1 week for each of the 7 participants) where app use abstracted from the backend was higher than self-reported app use.

For participants in the calorie tracking condition, research staff reviewed MyFitnessPal records, and we coded a *complete day* of calorie tracking any day in which participants tracked 2+ meals and 800+ kcal/day, as has been done elsewhere [20,21]. As participants in the calorie tracking condition were instructed to track all food and beverage intake, we only included *complete days* of tracking in our calculations of MyFitnessPal use.

Using the above definitions, we calculated the number and percentage of days participants in each treatment condition used their assigned app over the 12-week intervention. As the Slip Buddy app was down for 2 days in week 3, participants in this condition could have only used the app on a maximum of 82 days versus the 84 possible days for participants in the calorie tracking condition. We also categorized participants in both conditions as to whether they used their assigned app at least once over the 12-week intervention and whether they used their assigned app during week 12 as a measure of sustained engagement. Two participants were withdrawn or dropped out of the intervention because of incident pregnancies. For these women, app use was not assessed after they were no longer in the intervention (after week 7 for the Slip Buddy participant who became pregnant and after week 2 for the calorie tracking participant); instead, the calculation of percentage of days the app was used only counted days they were in the intervention.

### Slip Buddy App Data (Slip Buddy Participants Only)

#### Slips Reported

Backend data from the app were used to describe the number of slips reported for each participant during the intervention period and contextual factors related to slips.

#### Location of Slip

Participants were asked to enter a note about where they were when the slip occurred. The first author collapsed responses into categories that included work, home, other person’s house, restaurant/bar, at an event (eg, football game), in the car, or at the gym.

#### Nature of Eating Episode

Participants also indicated the nature of the eating episode in which the slip occurred, which included the choices of breakfast, lunch, dinner, dessert, snack, or alcohol. Alcohol was included to capture drinking episodes that happen outside of the context of meals or snacks and to prompt participants to think of excess alcohol intake as a dietary slip.

#### Activity During Slip

Participants also indicated what they were doing from a drop-down menu of domestic activities (eg, chores), working/studying (eg, employment and school), socializing, screen time, or commuting.

#### Stress and Hunger or Fullness Ratings of Slips

When they entered a slip, participants rated how much stress they were experiencing before their slip on a scale of 0 to 10, where 0 indicates no stress and 10 indicates extreme stress. Stress scores of 5 and above were considered moderate to high stress, whereas stress ratings of less than 5 were considered low stress. Participants also rated how hungry or full they felt before they slipped on a scale of 0 to 10, where 0 indicates extremely hungry, 5 indicates comfortably full, and 10 indicates stuffed, that is, uncomfortably full.

#### MyFitnessPal Data (Calorie Tracking Participants Only)

The participants were asked to record their diet every day for 12 weeks. These data were extracted from MyFitnessPal and coded for analysis. The first level of coding included recording each day that the participant entered at least one item. The second level extracted the number of eating episodes and calories each day.

#### Usability

The System Usability Scale (SUS) [22] was used at 12-weeks to assess the Slip Buddy app's usability. The SUS is a 10-item 5-point Likert scale questionnaire regarding human-computer interaction. For a participant who only answered 9 of the 10 questions, we used their mean of those 9 items to impute a response to the tenth item. An SUS score above 70 is considered acceptable and above average, whereas a score above 85 is considered excellent [23]. Moreover, when users rate a system with an SUS score of 82 (SD 5), they tend to be *promoters* of the system, which means they are likely to recommend it to a friend [24].

#### Acceptability

At 12 weeks, participants in both conditions rated the helpfulness and ease of use of their assigned app (response options: strongly disagree, disagree, neutral, agree, or strongly agree). We dichotomized responses as strongly agree/agree versus strongly disagree/disagree/neutral. As the Slip Buddy app is exclusively focused on diet, unlike calorie tracking apps that address both diet and exercise, we included a follow-up question asking participants to rate whether a feature that would allow them to track exercise slips (ie, times when they had planned to exercise but did not follow through) would increase the effectiveness of Slip Buddy app. Acceptability was also evaluated in postintervention focus groups via 2 questions: “what did you like most about Slip Buddy app and why?” and “what did you like least about Slip Buddy app and why?” During the intervention, participants started a discussion about the possibility of the Slip Buddy app having a feature that would allow people to track when they were tempted to slip but resisted that temptation. Given the enthusiasm for the idea, we added a question to the focus group script asking participants about the extent to which they would like to track temptations that did not turn into slips.

#### Burden

At 12 weeks, participants in both conditions rated how burdensome it was to use their assigned app on a scale of 0 to 100, with 0 being not at all burdensome and 100 being very burdensome. Participants rated how much they agreed that the app was time consuming, taxing, and tedious (response options: strongly disagree, disagree, neutral, agree, or strongly agree). We dichotomized responses as strongly agree/agree versus strongly disagree/disagree/neutral.

#### Weight

Weight was obtained at baseline and at 12 weeks via the Wi-Fi scales sent to participants upon enrollment.

#### Participation Engagement in the Facebook Group

Participant engagement is defined as participant posts, replies, reactions (eg, love, wow, angry, and sad), and participation in intervention polls, which are used either as a way of assessing participant knowledge (eg, pop quizzes) or as a way for participants to share their diet and/or exercise barriers. We extracted engagement data from the private Facebook group using the Grytics app, except poll data, which were manually extracted because Grytics does not capture poll data. We summarized the total number of original posts, replies, reactions, and polls that each participant participated in. In addition, we calculated the percentage of participants in each condition who replied to each of the 12 weekly weigh-in posts.

### Statistical Analysis

We summarized retention, app use, slips, usability, acceptability, burden, and engagement in the Facebook groups, including the percentage participating in the weekly weigh-ins using descriptive statistics. For variables that were normally distributed, we described distributions using mean and SD, and for variables that were not normally distributed, we described distributions using median and IQR. We compared use, retention, usability, acceptability, and engagement by treatment condition using *t* tests, chi-square tests, Fisher exact tests, or Mann-Whitney *U* test as appropriate. We compared the treatment conditions on app burden using the Wilcoxon rank-sum test. Insufficient retention, acceptability, and use were assumed if the calorie tracking condition showed a statistically significant advantage relative to Slip Buddy. Statistical tests were not used to compare groups on weight loss because this pilot study was not powered for weight loss efficacy; thus, it is not appropriate to perform such tests, as discussed elsewhere [25]. We used an intent-to-treat approach to describe the weight change. Two participants (1 in each condition) became pregnant during the study period. We used the latest available prepregnancy weights (from weeks 2 and 3 for the 2 participants, respectively) from their study scales as their follow-up values. Three participants did not provide weight during the follow-up. We also used their latest weight from their study scales (weeks 6, 9, and 10, for the 3 participants, respectively) as follow-up values. We secondarily reported weight loss assuming no weight loss for the 2 participants who became pregnant and the 3 participants lost to follow-up (ie, baseline observation carried forward), and we secondarily reported weight change excluding these 5 participants for whom we did not have nonpregnant follow-up weight. We conducted a directed content analysis [26] of the focus group data on acceptability. The first author developed a codebook based on themes emerging from the participant responses. Two coders independently coded responses, and discussion was used to achieve consensus on disagreements. Interrater reliability (IRR) was also calculated [27]. We summarized the frequency of the themes. Data management and quantitative analyses were conducted using SAS 9.4 (SAS Institute Inc).

## Results

### Recruitment

**Figure 2:**
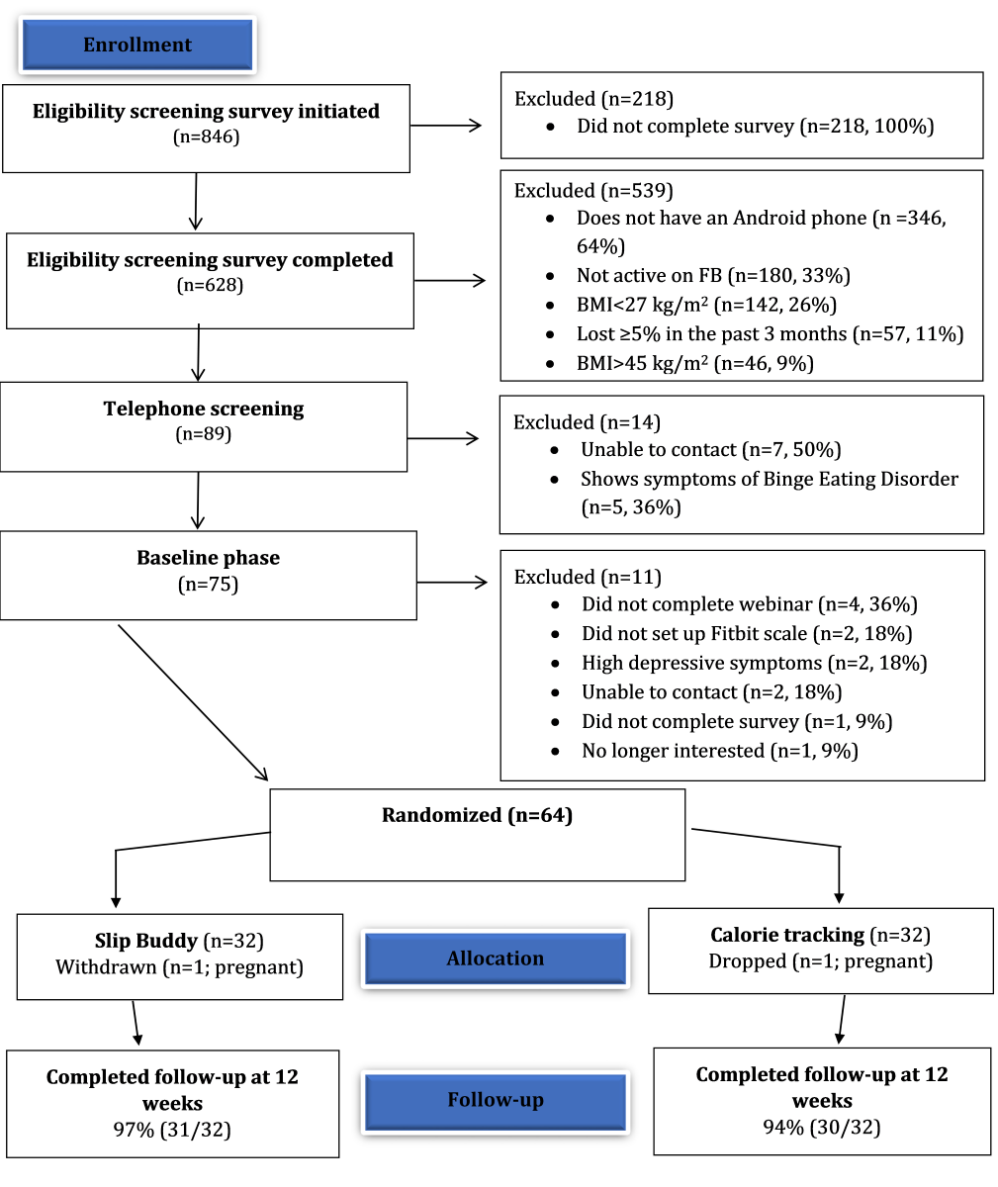
Consolidated Standards of Reporting Trials diagram. FB: Facebook.

A total of 846 individuals initiated the eligibility screening survey (Figure 2). Among individuals screened for eligibility, the most common reasons for exclusion were not owning an Android phone, not being an active Facebook user, BMI outside the eligible range, or recent weight losses of 5% or more (Figure 2). We randomized the 64 participants to 1 of 2 treatment conditions. Overall, participants were, on average, 39.8 (SD 11.0) years old, with a baseline BMI of 34.2 (SD 4.9) kg/m^2^; 75% (48/64) were female; and 81% (52/64) were non-Hispanic White. Participants lived in 18 US states, and 56% (36/64) of the participants were from Connecticut. Additional characteristics are presented in Table 1.

**Table 1:** Characteristics of participants by treatment condition (N=64).

Characteristics		Treatment condition	
		Slip Buddy (n=32)	Calorie tracking (n=32)
Age (years), mean (SD)		39.5 (9.6)	40.2 (12.3)
BMI at enrollment (kg/m^2^), mean (SD)		34.9 (5.3)	33.4 (4.4)
Female, n (%)		24 (75)	24 (75)
**Race, n (%)**			
	Non-Hispanic White	26 (81)	26 (81)
	Hispanic or Latino (any race)	1 (3)	4 (13)
	Non-Hispanic Black	3 (9)	1 (3)
	Non-Hispanic Asian	N/A^a^	1 (3)
	Other race or multiracial	2 (6)	N/A
**Marital status, n (%)**			
	Married or living with a partner	24 (75)	22 (69)
	Single	8 (25)	7 (22)
	Divorced or separated	N/A	3 (9)
**Education, n (%)**			
	At most high school	2 (6)	N/A
	Trade or technical school, some college, or associate degree	8 (25)	10 (31)
	Bachelor’s degree	9 (28)	4 (13)
	Some graduate coursework	4 (13)	7 (22)
	Graduate degree	9 (28)	11 (34)
**Employment status, n (%)^b^**			
	Employed full time	22 (69)	25 (78)
	Employed part time	8 (25)	5 (16)
	Homemaker (not looking for a job)	2 (6)	N/A
	Student	1 (3)	4 (13)
	Retired	N/A	1 (3)

^a^N/A: not applicable.

^b^Participants could select more than 1 employment status. In the Slip Buddy condition, 1 participant was employed part time and a student. In the calorie tracking condition, n=1 was employed part time and a student, n=1 was employed full time and a student, and n=1 was retired and employed part time.

### Retention

Retention was high in both treatment conditions, with 97% (31/32) of Slip Buddy participants and 94% (30/32) of calorie tracking participants providing follow-up data (*P*>.99, Fisher exact test).

### App Use

Nearly all participants randomized to the Slip Buddy condition (31/32, 97%) and the calorie tracking condition (31/32, 97%) used their assigned app at least once during the 12-week intervention. Participants in the Slip Buddy condition used their assigned app on a mean of 44.0 (SD 25.8) days. Participants in the calorie tracking condition used their app on a mean of 46.3 days (SD 24.0), which represented use on an average of 53.8% (SD 31.3%) of possible days for participants in the Slip Buddy condition and 57.5% (SD 28.4%) of possible days for participants in the calorie tracking condition (t_62_=0.495; *P*=.44). In terms of sustained use of their assigned app, 55% (18/31) of Slip Buddy participants used the app at least once in week 12 of the intervention compared with only 35% (11/31) of calorie tracking participants (*X*^2^_1_=2.4; *P*=.13). The proportion of participants using their assigned app each week of the 12-week intervention period is shown in Multimedia Appendix 1.

App use by Slip Buddy participants was nearly identical in a sensitivity analysis assessing app use by self-report data when available, and when self-report data were not available, backend data were used. Overall, 97% (30/31) of participants used the app at least once during the 12-week intervention. They used the app on an average of 43.8 (SD 25.7) days, representing 53.4% (SE 31.2%) of possible days; 55% (17/31) of the participants used the app at least once in week 12 of the intervention.

### Slips Reported

One participant did not use the Slip Buddy app, and 1 participant responded to the notification but did not record any slips. The remaining participants reported a total of 538 slips during the 12-week intervention period. Participants who reported slips (n=30) reported a median of 15 slips (IQR 8-23; range 2-66; mean 17.9, SD 14.4). Most of slips happened at home (297/538, 55.2%), followed by work (113/538, 21.0%) and restaurant/bar (86/538, 16.0%; Table 2). The nature of the eating episode most likely to be reported as a slip was a snack (220/538, 40.9%), followed by dinner (102/538, 19.0%), dessert (77/538, 14.3%), and lunch (60/538, 11.2%; Table 2). Activities engaged in when the slip occurred were split over work or studying (153/538, 28.4%), socializing (130/538, 24.2%), screen time (123/538, 22.9%), and domestic activities (112/538, 20.8%), and a small percentage of slips occurred while commuting (20/538, 3.7%; Table 2). The median stress rating during slips was 4 (IQR 2-5). The median hunger/fullness rating during slips was 4 (IQR 3-5). One-fifth of the slips (106/538, 20%) occurred when both stress and hunger were low, and another 20% (105/538) of the slips occurred when both stress and hunger were high.

**Table 2:** Location, activity, eating episode, stress, and satiety associated with slips reported by participants over 12 weeks (N=538 slips).

Slip characteristics			Value, n (%)
**Location**			
	Home	297 (55.2)	
	Work	113 (21)	
	Restaurant or bar	86 (16.0)	
	Another person’s house	22 (4.1)	
	The car	10 (1.9)	
	An event	8 (1.5)	
	The gym	2 (0.4)	
**Activity**			
	Work or studying	153 (28.4)	
	Socializing	130 (24.2)	
	Screen time	123 (22.9)	
	Domestic activities	112 (20.8)	
	Commuting	20 (3.7)	
**Eating episode**			
	Snack	220 (40.9)	
	Dinner	102 (19.0)	
	Dessert	77 (14.3)	
	Lunch	60 (11.2)	
	Breakfast	40 (7.4)	
	Alcohol	39 (7.3)	
**Stress**			
	Lower range, 0-4	298 (55.4)	
	Higher range, 5-10	240 (44.6)	
**Hunger to satiety**			
	Hungry range, 0-4	299 (55.6)	
	Full range, 5-10	239 (44.4)	

### Usability

The mean SUS score for the Slip Buddy condition was 64.8 (SD 16.5), which is the marginally acceptable range. Comparatively, the mean SUS score for participants in the calorie tracking condition was 76.3 (SD 17.6), which is considered good acceptability. Participants in the calorie tracking condition rated the MyFitnessPal app as more usable on average than the Slip Buddy participants rated the Slip Buddy app (t_59_=2.64; *P*=.01). Usability issues reported during the intervention included the Slip Buddy app crashing on some phone models and a temporary outage, both of which were fixed during the study.

### Acceptability

Among participants who completed the follow-up survey, 39% (12/31) of Slip Buddy participants agreed or strongly agreed that tracking slips was helpful, whereas 77% (23/30) of calorie tracking participants agreed or strongly agreed that tracking diet and exercise was helpful (*X*^2^_1,N=61_=8.9; *P*=.003; Table 3).

**Table 3:** Acceptability of assigned tracking app by treatment condition.

Acceptability item^a^	Slip Buddy (n=31), n (%)	Calorie tracking (n=30), n (%)	*X*^2^ (df)	*P* value
Tracking my slips with Slip Buddy app/tracking my diet and exercise with MyFitnessPal was helpful for me	12 (39)	23 (77)	8.9 (1)	.003
Tracking diet slips on Slip Buddy app/MyFitnessPal is easy	24 (77)	26 (87)	0.9 (1)	.35
Using the Slip Buddy app/MyFitnessPal is tedious	9 (29)	12 (40)	0.8 (1)	.37
Using the Slip Buddy app/MyFitnessPal is taxing	5 (16)	8 (28)	1.2 (1)	.28
Using the Slip Buddy app/MyFitnessPal is time consuming	6 (20)	13 (43)	3.8 (1)	.05

^a^Proportion of participants responding with *strongly agree* or *agree* versus *strongly disagree*, *disagree*, or *neutral*.

Most of both Slip Buddy (24/31, 77%) and calorie tracking participants (26/30, 87%) agreed or strongly agreed that using their respective app was easy (*X*^2^_1_=0.9; *P*=.37; Table 3). Two-thirds of Slip Buddy participants (21/31, 68%) agreed or strongly agreed that adding the ability to track exercise slips would be helpful.

### Burden

On a scale of 0 to 100, the median burden rating for participants in the Slip Buddy condition was 30 (IQR 15-50), and the median burden rating for participants in the calorie tracking condition was 45 (IQR 10-60; Mann-Whitney *U* test, *U*=949.5000; *P*=.78). The proportion of participants who agreed or strongly agreed that using their assigned app was tedious or taxing did not differ by treatment condition (Table 3). Finally, 20% (6/31) of Slip Buddy participants agreed or strongly agreed that using the Slip Buddy app was time consuming, whereas 43% (13/30) of MyFitnessPal participants agreed or strongly agreed that using MyFitnessPal was time consuming (*X*^2^_1_=3.8; *P*=.05; Table 2).

### Feedback From Participants in the Slip Buddy Condition

A total of 88% (28/32) of participants in the Slip Buddy condition attended postintervention focus groups, and they made a total of 35 responses to the question about what they liked most about the Slip Buddy app (IRR=94%; κ=0.89). The most common theme of responses was the ease of use/simple concept (23/28, 66%), followed by increasing accountability and/or awareness of overeating and/or triggers (8/28, 23%), the end-of-day reminder to track slips (2/28, 5%), feeling motivated to not slip so there would be nothing to track (1/28, 3%), and other (1/28, 3%). Participants made a total of 35 responses about what they liked least about the Slip Buddy app (IRR=91%; κ=0.89). Responses reflected the following themes: technical issues (eg, app crashing and notifications not going away; 10/35, 29%), easy to forget to use or not sure how to use when no slips (7/35, 21%), did not find relevant stress ratings (5/35, 15%), focus on slips was too negative (4/35, 12%), was not sure what to count as a slip (4/35, 12%), did not include diet instruction (4/35, 12%), and slip history screen was not as informative as it could be (1/35, 3%). For the final question regarding their thoughts on a feature that would allow them to track when they were tempted but did not slip, 75% (21/28) said they would be enthusiastic about this feature. The remainder said they worried that it would add too much burden.

### Weight Change

Over 12 weeks, participants randomized to the Slip Buddy condition had an average weight loss of −6.5 lb (SD 9.7) or 3.0% (SD 4.5%) of their baseline weight, and participants randomized to the calorie tracking condition had a weight loss of −7.5 lb (SD 10.7) or 3.6% (SD 4.9%) of their baseline weight (Table 4). In terms of clinically significant weight loss, 31% (10/32) and 34% (11/32) of participants randomized to the Slip Buddy and calorie tracking conditions, respectively, achieved 5% or greater weight loss, and 47% (15/32) and 47% (15/32), respectively, achieved 3% or greater weight loss (Table 4). In a secondary analysis assuming no weight loss for the 2 participants who became pregnant and the 3 participants lost to follow-up (ie, baseline observation carried forward approach), weight losses were on average −6.1 (SD 9.8) lb and −2.8% (SD 4.6%) among Slip Buddy participants and on average −7.1 (SD 10.8) lb and −3.4% (SD 5.0%) among calorie tracking participants, with 31% (10/32) and 34% (11/32) of participants, respectively, losing 5% or more weight compared with baseline, and 44% (14/32) and 44% (14/32), respectively, losing 3% or more weight compared with baseline. In a secondary analysis of the 59 participants who provided weight at follow-up and were not pregnant (n=30 Slip Buddy and n=29 calorie tracking), weight losses were on average −6.5 (SD 10.0) lb and −3.0% (SD 4.7%) among Slip Buddy participants and on average −7.9 (SD 11.1) lb and −3.8% (SD 5.1%) among calorie tracking participants, with 33% (10/30) and 38% (11/29) of participants, respectively, achieving 5% or greater weight loss from baseline and 47% (14/30) and 48% (14/29), respectively, achieving 3% or greater weight loss.

**Table 4:** Weight change from baseline to 12 weeks, by treatment condition.

Weight variables^a^	Slip Buddy (n=32)	Calorie tracking (n=32)
Baseline weight (lb)^b^, mean (SD)	217.2 (39.0)	208.5 (37.2)
Follow-up weight (lb), mean (SD)	210.7 (39.5)	201.0 (36.3)
Absolute weight change (lb), mean (SD)	−6.5 (9.7)	−7.5 (10.7)
Percentage weight change, mean (SD)	−3.0 (4.5)	−3.6 (4.9)
5% or greater weight loss, n (%)	10 (31)	11 (34)
3% or greater weight loss, n (%)	15 (47)	15 (47)

^a^We used the last available weight from the study scales for 8% (5/64) participants. In Slip Buddy condition, 3% (1/32) participant became pregnant (used week 3 weight), and 3% (1/32) participant did not provide follow-up weight (week 6 weight). In the calorie tracking condition, 3% (1/32) participant became pregnant (we used week 2 weight), and 6% (2/32) participants did not provide follow-up weight (we used weeks 9 and 10 weight).

^b^1 lb = 0.45 kg.

### Participant Engagement in the Facebook Group

In the Slip Buddy condition, the median total replies per participant was 55.00 (IQR 11.75-79.00), which was not significantly different from the median total replies of 29.5 (IQR 17.25-60.75; *U*=445.5000; *P*=.37) in the calorie tracking condition. In the Slip Buddy condition, participants reacted to a median of 13.00 (IQR 3.25-47.75) posts or replies, which was not significantly different from the median reactions of 13.00 (IQR 4.00-18.75; *U*=462.500; *P*=.51) in the calorie tracking condition. Few participants posted original posts (31% (10/32) in Slip Buddy and 34% (11/32) in calorie tracking); the median number of original posts participants made was 0 (IQR 0-1) in both conditions (*U*=508.000; *P*=.95). Finally, the Slip Buddy condition participants had a median total poll votes of 12.00 (IQR 6.25-16.00) compared with 8 (IQR 6.25-13.75) in the calorie tracking condition, a difference that was not statistically significant (*U*=440.500; *P*=.33).

In terms of weekly weigh-in participation, on average, 58.07% (SD 14.32; range 34%-81%) of Slip Buddy participants and 51.30% (SD 18.48; range 25%-88%) of calorie tracking participants replied to weigh-in posts each week (*t*_1,22_=1.003; *P*=.33). Week 1 had the highest participation in both groups (26/32, 81% and 28/32, 88% in Slip Buddy and calorie tracking, respectively), and participation declined over time to 63% (20/32) and 56% (18/32), respectively, by week 6 and to 34% (11/32) and 25% (8/32), respectively, by week 12.

## Discussion

### Principal Findings

Findings revealed that although participants in both treatment conditions used their assigned apps on a similar percentage of intervention days (ie, 54% of days for participants in the Slip Buddy condition and 58% of days for participants in the calorie tracking condition), 55% (17/31) of Slip Buddy participants used the app at week 12 of the intervention compared with only 35% (11/31) of calorie tracking participants. However, this difference was not statistically significant. Less than one-third of Slip Buddy participants agreed that using the Slip Buddy app was tedious (9/31, 29%), taxing (5/31, 16%), or time consuming (6/31, 20%); 77% (24/31) agreed that tracking slips was easy; but only 39% (12/31) agreed that tracking their slips with the app was helpful, which was significantly lower than that in the calorie tracking app condition. Slip Buddy also received lower usability ratings than the commercial calorie tracking app, perhaps not surprisingly, as commercial apps are years ahead of Slip Buddy in user experience optimization. Slip Buddy participants reported barriers such as technical difficulties, forgetting to use the app if they had not slipped in a while, and finding the exclusive focus on slips to be too negative. Slip data revealed that most of the slips reported happened at home, followed by work, and that snacks and dinner time were the eating episodes at which slips were most likely to occur. Activities that co-occurred with slips were distributed fairly evenly across work, socializing, screen time, and domestic tasks. Less than half of the slips occurred under conditions of moderate to high stress and over half occurred while hungry.

The Slip Buddy app was designed to reduce dietary self-monitoring to possibly its simplest form by only necessitating the recording of aberrant eating episodes. Despite its simplicity, use rates in this study appeared fairly comparable with app use in the commercial calorie tracking app condition. Interestingly, a randomized trial that compared a commercial calorie tracking app (Calorie Counter by Fat Secret) with the lower-burden Meal Logger app, which allows users to track their diet by taking photos of what they eat [28], found that the calorie tracking app was used on more intervention days over 6 months than the less intensive photo app (41% vs 28% of intervention days). Research on the adoption of digital health innovations suggests that usefulness and ease of use are 2 major drivers of use; thus, improving the use rates of Slip Buddy may involve enhancing these factors [29]. Accordingly, we are currently using our qualitative findings to guide updates to the app that would improve its usefulness and ease of use. Positive subjective social norms, meaning the belief that other people are using and benefiting from the app, have also been found to affect the use of digital health innovations [30]. People wanting to lose weight are more likely to have been exposed to commercial calorie tracking apps as these apps have been on the market for years and have millions of users, which has not only created social norms around these apps but has also allowed much more time for the user experience to be optimized. Given the long-term dominance of calorie tracking apps in the commercial weight loss space, these apps may also shape user expectations about what a weight loss app should be, and this could influence how they feel about apps that do not include the features they have come to expect. Indeed, some participants commented in the Facebook group that they would love to have the ability to track both slips and calories, whereas others were glad not to be tracking calories. At follow-up, we asked Slip Buddy participants if they had used a calorie tracking app in the past, and 68% (21/31) of participants said they had. Ultimately, people seem to want choices and flexibility in their options. Given that fatigue can set in with any long-term self-monitoring strategy, the ability to change self-monitoring strategies over time might be optimal.

Our focus group data revealed that the infrequency in which the Slip Buddy app needed to be used (ie, only when a slip occurs) may have led some people to forget to use it. Slip Buddy participants, on average, only recorded an average of 1 to 2 lapses per week, which is far less than daily and possibly insufficient to capture enough calories to lose weight even if all slips were successfully eliminated; however, without calorie data, this is unknown. Studies using ecological momentary assessment to have people track dietary lapses have reported means ranging from 2.7 to 11.8 lapses per week [31]. A recent trial that combined a web-based weight loss program with lapse tracking reported on average 29.7 lapses per participant over 10 weeks for a rate of 2.9 lapses per week over 10 weeks [15]. In that trial, participants were following the WW plan, and lapses were defined as any eating that went over the individual’s point target for a meal or snack. Having slips tied to calorie and/or point goals may give users more guidance on how to identify lapses. Our goal was to help people identify lapses in the absence of traditional forms of dietary tracking, so they may need more specific guidance on how to identify lapses. Focus groups revealed that a few participants were not sure what to count as a lapse, which may suggest that our definition of a slip was too narrow or too narrowly interpreted to capture enough eating episodes that could be considered dietary lapses. Alternatively, participants might not count slips that they felt were somehow justified, such as overeating at dinner on a day when a meal was skipped or having an extra slice of cake on one’s birthday. Research is needed to determine users’ lapse tracking accuracy by comparing lapse tracking data with 24-hour dietary recalls. Such data would also reveal the type of eating episodes people perceive as lapses and inform instructions they are given for how to track dietary lapses. With a rate of only 1 to 2 lapses recorded each week, instructions for lapse recording could be modified to help people capture more of their eating episodes that could be made healthier, not only to facilitate more consistent use but also to increase weight loss. The terms *slip* and *lapse* may be too limiting. Instead, users could be guided to track any eating episode they think has room for improvement or be given a minimum limit of eating episodes to record and work on each week, regardless of whether they consider those episodes to be slips or not. Another possible explanation for our finding of fewer lapses recorded relative to Forman et al [15] is that in their study, participants received 6 notifications per day to remind them to enter data relating to lapse triggers. Additional reminders to track lapses may increase users’ awareness of their dietary lapses.

Most participants wanted the Slip Buddy app to include the ability to track instances when they resisted temptations to give them an opportunity to see improvement in their ability to deal with contextual factors that cue slips. Including temptation tracking might allow users to develop a more regular habit of monitoring their eating habits and the circumstances in which they make healthy and unhealthy choices. In another study, participants in a behavioral weight loss program were asked to record their temptations and lapses in a paper diary in the final week of the program. They found that temptations were more likely than lapses to be followed by coping behaviors, suggesting the value of having people record both [12]. By starting with a very simple app and using a user-centered approach, we can now add new features suggested by participants only to the point at which those features continue to add value without undue burden. Most participants were also in favor of adding a feature that would allow them to track when they had exercise slips (ie, skipped a planned workout). Our next iteration will allow users to track these things. As a minority of our participants felt that additional features could undermine the app's simplicity, a user-centered process that is mindful of individual differences in perceived user burden will be important when adding new features to this and any app.

Despite the vast literature on emotional eating [32], only 44.6% (240/538) of slips occurred under conditions of moderate to high stress, and 14% (4/28) of participants in the focus groups said they did not find the stress ratings relevant to them because they do not feel they are a stress eater. Stress is one of many circumstances that can cue nonhomeostatic eating, and in this sample, it did not appear to drive most of the slips. Interestingly, 19.5% (105/538) of slips recorded were under conditions of both low stress and low hunger. The OnTrack app study included a wider range of triggers for users to record, including tiredness, temptations, missed meals/snacks, socializing, television, negative interpersonal interaction, cognitive load, food cues, alcohol consumption, unhealthy food availability, and planning food intake in their slate of possible triggers [15]. To satisfy individual differences and minimize user burden, apps could allow users to customize the cues they want to track by giving them choices from a wide range of options, including both emotional and physical states (eg, boredom and pain). An alternative explanation for a few slips recorded under conditions of high stress could be that stress may cause people to forget about a diet lapse or to be less aware of a diet lapse. Further research should explore how people decide an eating episode is a lapse and whether that changes under different emotional or physiological circumstances. For example, a person who eats a large amount in response to being extremely hungry may not perceive this eating as a lapse because they were very hungry and felt eating that volume of food was necessary under the circumstances. Some studies have users record their emotional and physical states throughout the day, which allows for even higher precision insights into the relationships between these states and dietary lapses [15,33].

This pilot feasibility trial was not powered to detect group differences in weight loss, the primary outcome planned for the larger fully powered randomized trial. Statistical comparisons of clinical outcomes in pilot feasibility trials are also inappropriate because of the inflated risk of type 1 and type 2 errors [25]. However, weight losses can be considered in the context of other trials of technology-based weight loss interventions. Participants randomized to the Slip Buddy condition lost an average of 6.5 lb in 12 weeks with a retention rate of 97%, which is comparable with the Meal Logger trial discussed above, where participants receiving the photo app lost 4.8 lb at 6 weeks and 5.5 lb at 6 months with a 96% retention rate [28]. In a pilot trial of a podcast and social media-delivered intervention, participants lost 6.4 lb in 12 weeks with a retention rate of 85% [34]. In our pilot trial, 31% (10/32) and 34% (11/32) of participants in the Slip Buddy and calorie tracking conditions, respectively, lost clinically significant weight (≥5%). This is comparable with findings from a randomized trial (NCT01479062) of a completely automated weight loss intervention based on the DPP in which 35% of participants lost clinically significant weight at 6 months [35], although our trial was only 12 weeks long compared with 6 months in that study. The mean percentage of weight loss in the Slip Buddy condition (ie, 3%) also fell into the range observed in the OnTrack study (ie, 2.91%-4.65% depending on the version of WW used in combination with OnTrack), which had participants track WW points, dietary lapses, and 16 lapse triggers 6 times a day [15]. Our findings suggest that some people in the Slip Buddy condition successfully lost clinically significant weight, although they were tracking slips and not total calories. Dietary mobile apps are not likely a one-size-fits-all. Further research should explore individual differences that predict who will be successful using different approaches to dietary self-monitoring.

This study had several limitations. First, the sample size was not large enough to compare the 2 conditions for weight loss. However, the purpose of this work was to evaluate the feasibility and acceptability of the Slip Buddy app using a user-informed process to guide improvements to the technology before conducting a fully powered randomized trial. We chose a 12-week intervention length to allow us to gain user insights after a prolonged period of use, but this study does not provide information on tracking habits over more extended periods, such as whether slip tracking decreases over time as participants learn their triggers and slip less. Another limitation is that our ability to measure app use via backend data was hampered by the fact that some participants did not see or receive end-of-day notifications that, when clicked, would indicate whether no slip entries meant a slip-free day or nonuse. For this reason, we had to rely on self-report use data that are prone to biases because of forgetting or social desirability. This can be ameliorated in the next version, and the addition of temptation and exercise slip tracking will give the user more to do each day with the app, even in the absence of slips. Another limitation is that the Slip Buddy app was not operating for 2 days, which could have affected use in subsequent days to the extent that participants were frustrated by this. As it is a newly developed app, bugs and crashes are more common than commercial apps that have been around for many years, and this will certainly impact usability ratings. An additional limitation is that both Slip Buddy and MyFitnessPal allow users to track other behaviors that may impact weight (eg, exercise, sleep, and mood), and we know little about the use of these features and whether their use impacted outcomes. Finally, a limitation is that the sample overrepresented non-Hispanic White women (40/64, 63% of our sample). In future research, recruitment will need to limit enrollment of non-Hispanic White women, the population segment that too often comprises most of the weight loss trial samples [36], to no more than the proportion of the population they represent.

Although human counseling is associated with better outcomes in technology-delivered behavioral weight loss interventions [37], it is also the main expense and primary barrier to scalability; thus, digital health tools that execute behavioral strategies are needed to reduce the time spent by human counselors. The Slip Buddy app was designed not only to simplify dietary self-monitoring but also to help users identify and disrupt cue-behavior linkages, a process that is typically facilitated via counseling. Data from this study will guide the next iteration of the Slip Buddy app, which will balance the need for simplicity with the need for additional technology-delivered behavioral strategies beyond self-monitoring and feedback. These findings can also inform future studies on how technology can be leveraged to execute simpler forms of dietary self-monitoring, given the high burden associated with traditional calorie tracking apps.

## Appendix

Multimedia Appendix 1 Proportion of participants using their assigned app each week of the 12-week intervention, by treatment condition.

Multimedia Appendix 2 CONSORT-EHEALTH checklist (V 1.6.1).

## Abbreviations

DPP: Diabetes Prevention Program
IRR: interrater reliability
SUS: System Usability Scale
WW: Weight Watchers
