# Supplementary material for: Slip Buddy App for Weight Management: Randomized Feasibility Trial of a Dietary Lapse Tracking App
Source: JMIR Mhealth Uhealth. 2021 Apr 1;9(4):e24249. doi: 10.2196/24249 (PMC8050748; doi:10.2196/24249)
Supplement: Multimedia Appendix 1 [file mhealth_v9i4e24249_app1.docx]

# Slip Buddy App for Weight Management: Randomized Feasibility Trial of a Dietary Lapse Tracking App

Supplemental Results

App use each week of the intervention

The proportion of participants using their assigned app each week of the 12-week intervention period is shown in Figure S1. Participants who become pregnant (1 in each condition) were not included after their removal from the intervention.

Figure S1. Proportion of participants using their assigned app each week of the 12-week intervention, by treatment condition.
