# Supplementary material for: Slip Buddy App for Weight Management: Randomized Feasibility Trial of a Dietary Lapse Tracking App
Source: JMIR Mhealth Uhealth. 2021 Apr 1;9(4):e24249. doi: 10.2196/24249 (PMC8050748; doi:10.2196/24249)
Supplement: Multimedia Appendix 2 [file mhealth_v9i4e24249_app2.pdf]

CONSORT-EHEALTH (V 1.6.1) - Submission/Publication Form

The CONSORT-EHEALTH checklist is intended for authors of randomized trials evaluating web-based and Internet-based applications/interventions, including mobile interventions, electronic games (incl multiplayer games), social media, certain telehealth applications, and other interactive and/or networked electronic applications. Some of the items (e.g. all subitems under item 5 - description of the intervention) may also be applicable for other study designs.

The goal of the CONSORT EHEALTH checklist and guideline is to be
a) a guide for reporting for authors of RCTs,
b) to form a basis for appraisal of an ehealth trial (in terms of validity)

CONSORT-EHEALTH items/subitems are MANDATORY reporting items for studies published in the Journal of Medical Internet Research and other journals / scientific societies endorsing the checklist.

Items numbered 1., 2., 3., 4a., 4b etc are original CONSORT or CONSORT-NPT (non-pharmacologic treatment) items.
Items with Roman numerals (i., ii, iii, iv etc.) are CONSORT-EHEALTH extensions/clarifications.

As the CONSORT-EHEALTH checklist is still considered in a formative stage, we would ask that you also RATE ON A SCALE OF 1-5 how important/useful you feel each item is FOR THE PURPOSE OF THE CHECKLIST and reporting guideline (optional).

Mandatory reporting items are marked with a red *.
In the textboxes, either copy & paste the relevant sections from your manuscript into this form - please include any quotes from your manuscript in QUOTATION MARKS,
or answer directly by providing additional information not in the manuscript, or elaborating on why the item was not relevant for this study.

YOUR ANSWERS WILL BE PUBLISHED AS A SUPPLEMENTARY FILE TO YOUR PUBLICATION IN JMIR AND ARE CONSIDERED PART OF YOUR PUBLICATION (IF ACCEPTED).
Please fill in these questions diligently. Information will not be copyedited, so please use proper spelling and grammar, use correct capitalization, and avoid abbreviations.

DO NOT FORGET TO SAVE AS PDF _AND_ CLICK THE SUBMIT BUTTON SO YOUR ANSWERS ARE IN OUR DATABASE !!!

Citation Suggestion (if you append the pdf as Appendix we suggest to cite this paper in the caption):
Eysenbach G, CONSORT-EHEALTH Group
CONSORT-EHEALTH: Improving and Standardizing Evaluation Reports of Web-based and

Mobile Health Interventions
J Med Internet Res 2011;13(4):e126
URL: http://www.jmir.org/2011/4/e126/
doi: 10.2196/jmir.1923
PMID: 22209829

* Required

Your name *

First Last

Sherry Pagoto

Primary Affiliation (short), City, Country *

University of Toronto, Toronto, Canada

University of Connecticut, USA

Your e-mail address *

abc@gmail.com

sherry.pagoto@uconn.edu

Title of your manuscript *

Provide the (draft) title of your manuscript.

Slip Buddy App for Weight Management: A Randomized Feasibility Trial of A Dietary Lapse Tracking App

Name of your App/Software/Intervention *

If there is a short and a long/alternate name, write the short name first and add the long name in brackets.

Slip Buddy App

Evaluated Version (if any)

e.g. "V1", "Release 2017-03-01", "Version 2.0.27913"

V2

Language(s) *

What language is the intervention/app in? If multiple languages are available, separate by comma (e.g. "English, French")

English

URL of your Intervention Website or App

e.g. a direct link to the mobile app on app in appstore (itunes, Google Play), or URL of the website. If the intervention is a DVD or hardware, you can also link to an Amazon page.

https://mhealth.inchip.uconn.edu/

URL of an image/screenshot (optional)

Your answer

Accessibility *

Can an enduser access the intervention presently?

- ○ access is free and open
- ◉ access only for special usergroups, not open
- ○ access is open to everyone, but requires payment/subscription/in-app purchases
- ○ app/intervention no longer accessible
- ○ Other:

Primary Medical Indication/Disease/Condition *

e.g. "Stress", "Diabetes", or define the target group in brackets after the condition, e.g. "Autism (Parents of children with)", "Alzheimers (Informal Caregivers of)"

"obesity"

Primary Outcomes measured in trial *

comma-separated list of primary outcomes reported in the trial

retention, app use, usability, acceptability

Secondary/other outcomes

Are there any other outcomes the intervention is expected to affect?

slips reported, contextual factors reported at slips, weight change

Recommended "Dose" *

What do the instructions for users say on how often the app should be used?

- (selected) Approximately Daily
- Approximately Weekly
- Approximately Monthly
- Approximately Yearly
- "as needed"
- Other:

Approx. Percentage of Users (starters) still using the app as recommended after 3 months *

- (selected) unknown / not evaluated
- 0-10%
- 11-20%
- 21-30%
- 31-40%
- 41-50%
- 51-60%
- 61-70%
- 71%-80%
- 81-90%
- 91-100%
- Other:

Overall, was the app/intervention effective? *

- ( ) yes: all primary outcomes were significantly better in intervention group vs control
- ( ) partly: SOME primary outcomes were significantly better in intervention group vs control
- ( ) no statistically significant difference between control and intervention
- ( ) potentially harmful: control was significantly better than intervention in one or more outcomes
- ( ) inconclusive: more research is needed
- (•) Other: this was a feasibility trial

Article Preparation Status/Stage *

At which stage in your article preparation are you currently (at the time you fill in this form)

- ( ) not submitted yet - in early draft status
- ( ) not submitted yet - in late draft status, just before submission
- ( ) submitted to a journal but not reviewed yet
- (•) submitted to a journal and after receiving initial reviewer comments
- ( ) submitted to a journal and accepted, but not published yet
- ( ) published
- ( ) Other:

Journal *

If you already know where you will submit this paper (or if it is already submitted), please provide the journal name (if it is not JMIR, provide the journal name under "other")

- ( ) not submitted yet / unclear where I will submit this
- ( ) Journal of Medical Internet Research (JMIR)
- (•) JMIR mHealth and UHealth
- ( ) JMIR Serious Games
- ( ) JMIR Mental Health
- ( ) JMIR Public Health
- ( ) JMIR Formative Research
- ( ) Other JMIR sister journal
- ( ) Other:

Is this a full powered effectiveness trial or a pilot/feasibility trial? *

- (•) Pilot/feasibility
- ( ) Fully powered

Manuscript tracking number *

If this is a JMIR submission, please provide the manuscript tracking number under "other" (The ms tracking number can be found in the submission acknowledgement email, or when you login as author in JMIR. If the paper is already published in JMIR, then the ms tracking number is the four-digit number at the end of the DOI, to be found at the bottom of each published article in JMIR)

- ( ) no ms number (yet) / not (yet) submitted to / published in JMIR
- (•) Other: #24249

TITLE AND ABSTRACT

1a) TITLE: Identification as a randomized trial in the title

1a) Does your paper address CONSORT item 1a? *

I.e does the title contain the phrase "Randomized Controlled Trial"? (if not, explain the reason under "other")

- ( ) yes
- (•) Other: it is a feasibility trial

1a-i) Identify the mode of delivery in the title

Identify the mode of delivery. Preferably use “web-based” and/or “mobile” and/or “electronic game” in the title. Avoid ambiguous terms like “online”, “virtual”, “interactive”. Use “Internet-based” only if Intervention includes non-web-based Internet components (e.g. email), use “computer-based” or “electronic” only if offline products are used. Use “virtual” only in the context of “virtual reality” (3-D worlds). Use “online” only in the context of “online support groups”. Complement or substitute product names with broader terms for the class of products (such as “mobile” or “smart phone” instead of “iphone”), especially if the application runs on different platforms.

	1	2	3	4	5	
subitem not at all important	( )	( )	( )	( )	(•)	essential

Clear selection

Does your paper address subitem 1a-i? *

Copy and paste relevant sections from manuscript title (include quotes in quotation marks "like this" to indicate direct quotes from your manuscript), or elaborate on this item by providing additional information not in the ms, or briefly explain why the item is not applicable/relevant for your study

Slip Buddy App for Weight Management: A Randomized Feasibility Trial of A Dietary Lapse Tracking App

1a-ii) Non-web-based components or important co-interventions in title

Mention non-web-based components or important co-interventions in title, if any (e.g., “with telephone support”).

	1	2	3	4	5	
subitem not at all important	(●)	( )	( )	( )	( )	essential

Clear selection

Does your paper address subitem 1a-ii?

Copy and paste relevant sections from manuscript title (include quotes in quotation marks "like this" to indicate direct quotes from your manuscript), or elaborate on this item by providing additional information not in the ms, or briefly explain why the item is not applicable/relevant for your study

Your answer

1a-iii) Primary condition or target group in the title

Mention primary condition or target group in the title, if any (e.g., “for children with Type I Diabetes”) Example: A Web-based and Mobile Intervention with Telephone Support for Children with Type I Diabetes: Randomized Controlled Trial

	1	2	3	4	5	
subitem not at all important	( )	( )	( )	( )	(●)	essential

Clear selection

Does your paper address subitem 1a-iii? *

Copy and paste relevant sections from manuscript title (include quotes in quotation marks "like this" to indicate direct quotes from your manuscript), or elaborate on this item by providing additional information not in the ms, or briefly explain why the item is not applicable/relevant for your study

Slip Buddy App for Weight Management: A Randomized Feasibility Trial of A Dietary Lapse Tracking App

1b) ABSTRACT: Structured summary of trial design, methods, results, and conclusions

NPT extension: Description of experimental treatment, comparator, care providers, centers, and blinding status.

1b-i) Key features/functionalities/components of the intervention and comparator in the METHODS section of the ABSTRACT

Mention key features/functionalities/components of the intervention and comparator in the abstract. If possible, also mention theories and principles used for designing the site. Keep in mind the needs of systematic reviewers and indexers by including important synonyms. (Note: Only report in the abstract what the main paper is reporting. If this information is missing from the main body of text, consider adding it)

	1	2	3	4	5	
subitem not at all important	○	○	○	○	◉	essential

Clear selection

Does your paper address subitem 1b-i? *

Copy and paste relevant sections from the manuscript abstract (include quotes in quotation marks "like this" to indicate direct quotes from your manuscript), or elaborate on this item by providing additional information not in the ms, or briefly explain why the item is not applicable/relevant for your study

"We designed Slip Buddy App as a possibly less burdensome form of dietary self-monitoring that allows users to track dietary lapses and the contextual factors surrounding them."

1b-ii) Level of human involvement in the METHODS section of the ABSTRACT

Clarify the level of human involvement in the abstract, e.g., use phrases like “fully automated” vs. “therapist/nurse/care provider/physician-assisted” (mention number and expertise of providers involved, if any). (Note: Only report in the abstract what the main paper is reporting. If this information is missing from the main body of text, consider adding it)

	1	2	3	4	5	
subitem not at all important	○	○	○	○	◉	essential

Clear selection

Does your paper address subitem 1b-ii?

Copy and paste relevant sections from the manuscript abstract (include quotes in quotation marks "like this" to indicate direct quotes from your manuscript), or elaborate on this item by providing additional information not in the ms, or briefly explain why the item is not applicable/relevant for your study

"We conducted a pilot randomized trial to evaluate the feasibility and acceptability of using Slip Buddy App compared to a popular commercial calorie tracking app during a counselor-delivered online behavioral weight loss intervention."

1b-iii) Open vs. closed, web-based (self-assessment) vs. face-to-face assessments in the METHODS section of the ABSTRACT

Mention how participants were recruited (online vs. offline), e.g., from an open access website or from a clinic or a closed online user group (closed usergroup trial), and clarify if this was a purely web-based trial, or there were face-to-face components (as part of the intervention or for assessment). Clearly say if outcomes were self-assessed through questionnaires (as common in web-based trials). Note: In traditional offline trials, an open trial (open-label trial) is a type of clinical trial in which both the researchers and participants know which treatment is being administered. To avoid confusion, use “blinded” or “unblinded” to indicated the level of blinding instead of “open”, as “open” in web-based trials usually refers to “open access” (i.e. participants can self-enrol). (Note: Only report in the abstract what the main paper is reporting. If this information is missing from the main body of text, consider adding it)

	1	2	3	4	5	
subitem not at all important	○	○	◉	○	○	essential

Clear selection

Does your paper address subitem 1b-iii?

Copy and paste relevant sections from the manuscript abstract (include quotes in quotation marks "like this" to indicate direct quotes from your manuscript), or elaborate on this item by providing additional information not in the ms, or briefly explain why the item is not applicable/relevant for your study

"Adults who were overweight or obese were recruited online and randomized into a 12-week online weight loss intervention that included either the Slip Buddy App or a commercial calorie tracking app."

1b-iv) RESULTS section in abstract must contain use data

Report number of participants enrolled/assessed in each group, the use/uptake of the intervention (e.g., attrition/adherence metrics, use over time, number of logins etc.), in addition to primary/secondary outcomes. (Note: Only report in the abstract what the main paper is reporting. If this information is missing from the main body of text, consider adding it)

	1	2	3	4	5	
subitem not at all important	○	○	○	○	◉	essential

Clear selection

Does your paper address subitem 1b-iv?

Copy and paste relevant sections from the manuscript abstract (include quotes in quotation marks "like this" to indicate direct quotes from your manuscript), or elaborate on this item by providing additional information not in the ms, or briefly explain why the item is not applicable/relevant for your study

"Participants (N=64) were 75% female and on average 39.8 (SD: 11.0) years old with mean BMI of 34.2 (SD: 4.9) kg/m2. Retention was high in both conditions, with 97% (n=31) retained in the Slip Buddy condition and 94% (n=30) retained in the calorie tracking condition. On average, participants used the Slip Buddy App on 53.8% of days (SD: 31.3%) which was not significantly different from the calorie tracking condition (57.5%, SD: 28.4, and participants who recorded slips (n=30; 94%) logged on average 17.9 (SD: 14.4) slips in 12 weeks."

1b-v) CONCLUSIONS/DISCUSSION in abstract for negative trials

Conclusions/Discussions in abstract for negative trials: Discuss the primary outcome - if the trial is negative (primary outcome not changed), and the intervention was not used, discuss whether negative results are attributable to lack of uptake and discuss reasons. (Note: Only report in the abstract what the main paper is reporting. If this information is missing from the main body of text, consider adding it)

	1	2	3	4	5	
subitem not at all important	(•)	( )	( )	( )	( )	essential

Clear selection

Does your paper address subitem 1b-v?

Copy and paste relevant sections from the manuscript abstract (include quotes in quotation marks "like this" to indicate direct quotes from your manuscript), or elaborate on this item by providing additional information not in the ms, or briefly explain why the item is not applicable/relevant for your study

NA for a feasibility trial

INTRODUCTION

2a) In INTRODUCTION: Scientific background and explanation of rationale

2a-i) Problem and the type of system/solution

Describe the problem and the type of system/solution that is object of the study: intended as stand-alone intervention vs. incorporated in broader health care program? Intended for a particular patient population? Goals of the intervention, e.g., being more cost-effective to other interventions, replace or complement other solutions? (Note: Details about the intervention are provided in “Methods” under 5)

	1	2	3	4	5	
subitem not at all important	○	○	○	○	◉	essential

Clear selection

Does your paper address subitem 2a-i? *

Copy and paste relevant sections from the manuscript (include quotes in quotation marks "like this" to indicate direct quotes from your manuscript), or elaborate on this item by providing additional information not in the ms, or briefly explain why the item is not applicable/relevant for your study

"Obesity is a major risk factor for type 2 diabetes[1] but many people still do not have access to evidence-based lifestyle interventions.[2] Technology-delivered lifestyle interventions can increase reach and early studies reveal promising impact, but they are still fairly burdensome and expensive.[3]"

2a-ii) Scientific background, rationale: What is known about the (type of) system

Scientific background, rationale: What is known about the (type of) system that is the object of the study (be sure to discuss the use of similar systems for other conditions/diagnoses, if appropiate), motivation for the study, i.e. what are the reasons for and what is the context for this specific study, from which stakeholder viewpoint is the study performed, potential impact of findings [2]. Briefly justify the choice of the comparator.

	1	2	3	4	5	
subitem not at all important	○	○	○	○	◉	essential

Clear selection

Does your paper address subitem 2a-ii? *

Copy and paste relevant sections from the manuscript (include quotes in quotation marks "like this" to indicate direct quotes from your manuscript), or elaborate on this item by providing additional information not in the ms, or briefly explain why the item is not applicable/relevant for your study

"A key source of burden is self-monitoring in the form of calorie tracking which requires a person to record all of the food and beverage they consume each day. While adherence to calorie tracking predicts weight loss outcomes,[4] adherence is notoriously low.[5] A recent study [NCT03254953] found that rates of consistent calorie tracking in an online weight loss program fell from 68% in week 1 to 21% by week 12.[6] Interventions that do not use calorie tracking have failed to produce weight loss outcomes,[7] thus new forms of self-monitoring that can produce similar or greater weight loss are needed.

Interestingly, calorie tracking is a relatively complex form of self-monitoring compared to forms used for other behaviors. For example, in smoking cessation interventions the smoker keeps a tally of the number of cigarettes smoked each day and notes the triggers associated with each smoking episode.[8] Similarly, a simpler way to perform dietary self-monitoring could be to have people track only the segment of their diet that requires intervention, i.e., the eating episodes that account for excess calories, or dietary lapses. Dietary lapses are considered “non-homeostatic eating” which includes any eating episode that occurs in excess of one’s needs to maintain health, not just in terms of food quantity but also food quality.[9] To the extent that we can help people identify and eliminate dietary lapses, we may be able to affect energy balance without the task of calorie tracking.

Previous ecological momentary assessment studies reveal that people are able to identify and self-monitor dietary lapses.[10-13] Carels and colleagues had 12 dieters track their dietary lapses over the course of the week along with contextual factors surrounding the lapses. Results revealed that hunger, location, negative affect, and certain activities such as watching TV and socializing were common during lapses. Stress, hunger, and socializing were found to be predictors of dietary lapses in other studies as well.[13, 14] This research has stimulated at least two dietary lapse tracking apps designed for use in behavioral weight loss interventions [15, 16]. OnTrack allows users to track their dietary lapses from the WW (formerly Weight Watchers) online points-based weight loss program and the contextual factors surrounding those lapses. Six prompts per day ask the users to record 17 contextual factors (e.g., mood, hunger, temptations), the data from which goes into a machine learning algorithm that drives just-in-time intervention messages that occur when the user is at high risk for lapses.[10, 15] In a randomized trial, adding OnTrack to WW (formerly Weight Watchers) points based weight loss program was [illegible]

found superior in weight loss outcomes to the WW program alone over 10 weeks, but did not appear to enhance weight loss when added to a version of the WW program that was less intensive in terms of point tracking.[15] In contrast to OnTrack which requires users to do WW point tracking, the Slip Buddy app was designed as a replacement for traditional forms of dietary tracking."

2b) In INTRODUCTION: Specific objectives or hypotheses

Does your paper address CONSORT subitem 2b? *

Copy and paste relevant sections from the manuscript (include quotes in quotation marks "like this" to indicate direct quotes from your manuscript), or elaborate on this item by providing additional information not in the ms, or briefly explain why the item is not applicable/relevant for your study

"This study is a pilot feasibility randomized controlled trial in which participants with overweight or obesity were randomized to receive either the Slip Buddy App or a commercial calorie tracking app during a 12 week counselor-led online weight loss intervention. Our first aim was to compare groups on retention and app usage to explore whether the commercial app is significantly superior to Slip Buddy which would point to the need for further modification to Slip Buddy before proceeding to a fully powered efficacy trial. Our second aim was to describe the total number of slips reported and the contextual factors reported at slips including location of slips, type of eating episode (e.g., lunch, snack), stress, and hunger/satiety. This aim was descriptive in nature. Our third aim was to assess the usability, acceptability, and burden of Slip Buddy App quantitatively and via qualitative interviews where participants shared what they liked and disliked about Slip Buddy App and features they would like added. Like aim 1, we tested whether the commercial app is significantly superior in usability, acceptability, and burden which would signal areas for further modification to Slip Buddy before proceeding to an efficacy trial. Our fourth aim was to describe percent weight loss from baseline to 12 weeks in both groups, including the proportion of participants who lost clinically significant weight. This aim is exploratory because only a fully powered efficacy trial could address this question and that is premature during this developmental stage. "

METHODS

3a) Description of trial design (such as parallel, factorial) including allocation ratio

Does your paper address CONSORT subitem 3a? *

Copy and paste relevant sections from the manuscript (include quotes in quotation marks "like this" to indicate direct quotes from your manuscript), or elaborate on this item by providing additional information not in the ms, or briefly explain why the item is not applicable/relevant for your study

We conducted a pilot feasibility randomized trial in which participants who are overweight or obese were recruited into a remotely delivered intervention via online advertisements at the University of Connecticut, on ResearchMatch, and in Facebook groups across the US between July and October of 2019.

3b) Important changes to methods after trial commencement (such as eligibility criteria), with reasons

Does your paper address CONSORT subitem 3b? *

Copy and paste relevant sections from the manuscript (include quotes in quotation marks "like this" to indicate direct quotes from your manuscript), or elaborate on this item by providing additional information not in the ms, or briefly explain why the item is not applicable/relevant for your study

"We conducted a pilot feasibility randomized trial in which participants who are overweight or obese were recruited into a remotely delivered intervention via online advertisements at the University of Connecticut, on ResearchMatch, and in Facebook groups across the US between July and October of 2019. "

3b-i) Bug fixes, Downtimes, Content Changes

Bug fixes, Downtimes, Content Changes: ehealth systems are often dynamic systems. A description of changes to methods therefore also includes important changes made on the intervention or comparator during the trial (e.g., major bug fixes or changes in the functionality or content) (5-iii) and other “unexpected events” that may have influenced study design such as staff changes, system failures/downtimes, etc. [2].

	1	2	3	4	5	
subitem not at all important	○	○	○	○	◉	essential

Clear selection

Does your paper address subitem 3b-i?

Copy and paste relevant sections from the manuscript (include quotes in quotation marks "like this" to indicate direct quotes from your manuscript), or elaborate on this item by providing additional information not in the ms, or briefly explain why the item is not applicable/relevant for your study

"As the nature of diet tracking differed between the two treatment conditions, how we assessed app use also differed. For participants in the Slip Buddy condition, we intended to categorize participants as having used the Slip Buddy App for a given day if (1) backend data from the app revealed at least one slip was recorded or check-in completed (optional), or (2) in the absence of slips, the participants responded to the end-of-day check-in saying that they did not have any slips that day. Staff reviewed the data in the Slip Buddy database server (i.e., backend data) and recorded the number of days each week each participant used the app (e.g., either recorded a slip or responded to a notification or check-in reporting that they experienced no slips). However, some participants reported that they did not see or receive the end-of-day check-in notifications from the app which we determined was related to the authentication token on the phone expiring periodically. The end-of-the-day notification gives the participant the opportunity to confirm that no slips occurred if none had been recorded thus far. Without the notification we cannot distinguish between a day in which the participant did not track slips and a day in which no slips occurred. For this reason, backend data would be an underestimation of app use. Because failure can sometimes happen while transmitting app data to the remote database server, we also collected self-report app use data by emailing participants each week a single item asking them how many days they used the app to track slips that week. Self-report data was available for 74% (n=282/379) of weeks across all participants (counting only 7 weeks for the participant who withdrew due to pregnancy). Because 26% of self-report data was missing and backend data was incomplete by an unknown amount, we leveraged both forms of data to measure app use. We used the larger of the two values for two reasons: 1) when self-report data is higher than backend data it could correct for the underestimation bias of backend data, and 2) when backend data is higher than self-report it could correct for recall bias from self-report. The weakness is that we don’t have a way to correct for recall bias from self-report that overestimates use which surely exists to some extent. Self-report data were used on 58% of total weeks (220 weeks) and backend data were used 27% of weeks (104 weeks) which includes the 7 weeks backend was higher than self-report and the 97 weeks in which self-report was missing. On the remaining 15% of weeks (55 weeks) self-report and backend were the same so that value was used. Even though there is no way to correct for possible overestimations via self-report, on 26% of weeks (97 weeks) only backend data were available which would be an underestimate for those weeks."

4a) Eligibility criteria for participants

Does your paper address CONSORT subitem 4a? *

Copy and paste relevant sections from the manuscript (include quotes in quotation marks "like this" to indicate direct quotes from your manuscript), or elaborate on this item by providing additional information not in the ms, or briefly explain why the item is not applicable/relevant for your study

"We recruited people interested in losing weight with BMI between 27-45 kg/m2, aged 18-65 years, who had an Android smartphone, and phone connectivity at home and work. Exclusion criteria were inability to walk unaided for ¼ mile without stopping, not a daily Facebook user (because the group-based part of the intervention was delivered via Facebook), taking medications known to affect appetite and/or weight, has a condition that precludes dietary changes (e.g., ulcerative colitis), type 1 or 2 diabetes, had gastric bypass surgery or plans to during the study period, pregnant or lactating, severe mental illness or substance use disorder, binge eating disorder, or lost 5% or more weight in the past 3 months."

4a-i) Computer / Internet literacy

Computer / Internet literacy is often an implicit “de facto” eligibility criterion - this should be explicitly clarified.

	1	2	3	4	5	
subitem not at all important	○	○	○	○	◉	essential

Clear selection

Does your paper address subitem 4a-i?

Copy and paste relevant sections from the manuscript (include quotes in quotation marks "like this" to indicate direct quotes from your manuscript), or elaborate on this item by providing additional information not in the ms, or briefly explain why the item is not applicable/relevant for your study

"We recruited people who had an Android smartphone, and phone connectivity at home and work. Exclusion criteria were inability to walk unaided for ¼ mile without stopping, not a daily Facebook user (because the group-based part of the intervention was delivered via Facebook)."

4a-ii) Open vs. closed, web-based vs. face-to-face assessments:

Open vs. closed, web-based vs. face-to-face assessments: Mention how participants were recruited (online vs. offline), e.g., from an open access website or from a clinic, and clarify if this was a purely web-based trial, or there were face-to-face components (as part of the intervention or for assessment), i.e., to what degree got the study team to know the participant. In online-only trials, clarify if participants were quasi-anonymous and whether having multiple identities was possible or whether technical or logistical measures (e.g., cookies, email confirmation, phone calls) were used to detect/prevent these.

	1	2	3	4	5	
subitem not at all important	○	○	○	○	◉	essential

Clear selection

Does your paper address subitem 4a-ii? *

Copy and paste relevant sections from the manuscript (include quotes in quotation marks "like this" to indicate direct quotes from your manuscript), or elaborate on this item by providing additional information not in the ms, or briefly explain why the item is not applicable/relevant for your study

"We conducted a pilot feasibility randomized trial in which participants who are overweight or obese were recruited into a remotely delivered intervention via online advertisements at the University of Connecticut, on ResearchMatch, and in Facebook groups across the US between July and October of 2019."

4a-iii) Information giving during recruitment

Information given during recruitment. Specify how participants were briefed for recruitment and in the informed consent procedures (e.g., publish the informed consent documentation as appendix, see also item X26), as this information may have an effect on user self-selection, user expectation and may also bias results.

	1	2	3	4	5	
subitem not at all important	○	○	○	○	◉	essential

Clear selection

Does your paper address subitem 4a-iii?

Copy and paste relevant sections from the manuscript (include quotes in quotation marks "like this" to indicate direct quotes from your manuscript), or elaborate on this item by providing additional information not in the ms, or briefly explain why the item is not applicable/relevant for your study

"Prior to randomization potential participants were required to attend an orientation webinar in which study staff used a methods-motivational interviewing approach to help them understand the scientific rationale of the trial design, research questions, and methods. This helps participants understand the commitment entailed in trial enrollment and helps set clear expectations (e.g., transparency about length of assessments), explain the scientific rationale for procedures (e.g., randomization, feasibility versus efficacy testing), diffuse ambivalence about research participation using motivational interviewing techniques, and make explicit commitments to self and trial methods. Upon completion, those interested in proceeding with the study were mailed a wifi scale (Fitbit Aria) and asked to provide staff with login info for the scale so that weight could be recorded for assessments. After randomization, participants had a 60-minute call with a study staff person to receive guidance on how to download and use their assigned app and be entered into their assigned Facebook group. Participants were allowed to keep the scale and were compensated for completing assessments."

4b) Settings and locations where the data were collected

Does your paper address CONSORT subitem 4b? *

Copy and paste relevant sections from the manuscript (include quotes in quotation marks "like this" to indicate direct quotes from your manuscript), or elaborate on this item by providing additional information not in the ms, or briefly explain why the item is not applicable/relevant for your study

"Participants in both conditions were assigned one of the diet tracking apps and received the Diabetes Prevention Program (DPP) lifestyle intervention delivered within a counselor-led private Facebook group that included all participants in their respective condition."

4b-i) Report if outcomes were (self-)assessed through online questionnaires

Clearly report if outcomes were (self-)assessed through online questionnaires (as common in web-based trials) or otherwise.

	1	2	3	4	5	
subitem not at all important	○	○	○	○	◉	essential

Clear selection

Does your paper address subitem 4b-i? *

Copy and paste relevant sections from the manuscript (include quotes in quotation marks "like this" to indicate direct quotes from your manuscript), or elaborate on this item by providing additional information not in the ms, or briefly explain why the item is not applicable/relevant for your study

"Participants eligible after the screening survey were emailed the consent form and completed a telephone screening call. The screening call included a review of the consent form, any remaining eligibility-related questions, and an emailed link to the baseline survey. Weight was obtained at baseline and 12 weeks from the wi-fi scales sent to participants upon enrollment."

4b-ii) Report how institutional affiliations are displayed

Report how institutional affiliations are displayed to potential participants [on ehealth media], as affiliations with prestigious hospitals or universities may affect volunteer rates, use, and reactions with regards to an intervention.(Not a required item – describe only if this may bias results)

	1	2	3	4	5	
subitem not at all important	◉	○	○	○	○	essential

Clear selection

Does your paper address subitem 4b-ii?

Copy and paste relevant sections from the manuscript (include quotes in quotation marks "like this" to indicate direct quotes from your manuscript), or elaborate on this item by providing additional information not in the ms, or briefly explain why the item is not applicable/relevant for your study

Your answer

5) The interventions for each group with sufficient details to allow replication, including how and when they were actually administered

5-i) Mention names, credential, affiliations of the developers, sponsors, and owners

Mention names, credential, affiliations of the developers, sponsors, and owners [6] (if authors/evaluators are owners or developer of the software, this needs to be declared in a “Conflict of interest” section or mentioned elsewhere in the manuscript).

	1	2	3	4	5	
subitem not at all important	◉	○	○	○	○	essential

Clear selection

Does your paper address subitem 5-i?

Copy and paste relevant sections from the manuscript (include quotes in quotation marks "like this" to indicate direct quotes from your manuscript), or elaborate on this item by providing additional information not in the ms, or briefly explain why the item is not applicable/relevant for your study

Your answer

5-ii) Describe the history/development process

Describe the history/development process of the application and previous formative evaluations (e.g., focus groups, usability testing), as these will have an impact on adoption/use rates and help with interpreting results.

	1	2	3	4	5	
subitem not at all important	○	○	○	○	◉	essential

Clear selection

Does your paper address subitem 5-ii?

Copy and paste relevant sections from the manuscript (include quotes in quotation marks "like this" to indicate direct quotes from your manuscript), or elaborate on this item by providing additional information not in the ms, or briefly explain why the item is not applicable/relevant for your study

"Employing user-centered design, we developed the Slip Buddy App which has users track dietary lapses as they occur and the contextual factors that cued each lapse.[16]"

5-iii) Revisions and updating

Revisions and updating. Clearly mention the date and/or version number of the application/intervention (and comparator, if applicable) evaluated, or describe whether the intervention underwent major changes during the evaluation process, or whether the development and/or content was “frozen” during the trial. Describe dynamic components such as news feeds or changing content which may have an impact on the replicability of the intervention (for unexpected events see item 3b).

	1	2	3	4	5	
subitem not at all important	(●)	( )	( )	( )	( )	essential

Clear selection

Does your paper address subitem 5-iii?

Copy and paste relevant sections from the manuscript (include quotes in quotation marks "like this" to indicate direct quotes from your manuscript), or elaborate on this item by providing additional information not in the ms, or briefly explain why the item is not applicable/relevant for your study

Your answer

5-iv) Quality assurance methods

Provide information on quality assurance methods to ensure accuracy and quality of information provided [1], if applicable.

	1	2	3	4	5	
subitem not at all important	(●)	( )	( )	( )	( )	essential

Clear selection

Does your paper address subitem 5-iv?

Copy and paste relevant sections from the manuscript (include quotes in quotation marks "like this" to indicate direct quotes from your manuscript), or elaborate on this item by providing additional information not in the ms, or briefly explain why the item is not applicable/relevant for your study

Your answer

5-v) Ensure replicability by publishing the source code, and/or providing screenshots/screen-capture video, and/or providing flowcharts of the algorithms used

Ensure replicability by publishing the source code, and/or providing screenshots/screen-capture video, and/or providing flowcharts of the algorithms used. Replicability (i.e., other researchers should in principle be able to replicate the study) is a hallmark of scientific reporting.

	1	2	3	4	5	
subitem not at all important	◉	○	○	○	○	essential

Clear selection

Does your paper address subitem 5-v?

Copy and paste relevant sections from the manuscript (include quotes in quotation marks "like this" to indicate direct quotes from your manuscript), or elaborate on this item by providing additional information not in the ms, or briefly explain why the item is not applicable/relevant for your study

Your answer

5-vi) Digital preservation

Digital preservation: Provide the URL of the application, but as the intervention is likely to change or disappear over the course of the years; also make sure the intervention is archived (Internet Archive, webcitation.org, and/or publishing the source code or screenshots/videos alongside the article). As pages behind login screens cannot be archived, consider creating demo pages which are accessible without login.

	1	2	3	4	5	
subitem not at all important	○	○	○	○	◉	essential

Clear selection

Does your paper address subitem 5-vi?

Copy and paste relevant sections from the manuscript (include quotes in quotation marks "like this" to indicate direct quotes from your manuscript), or elaborate on this item by providing additional information not in the ms, or briefly explain why the item is not applicable/relevant for your study

screenshots are provided

5-vii) Access

Access: Describe how participants accessed the application, in what setting/context, if they had to pay (or were paid) or not, whether they had to be a member of specific group. If known, describe how participants obtained “access to the platform and Internet” [1]. To ensure access for editors/reviewers/readers, consider to provide a “backdoor” login account or demo mode for reviewers/readers to explore the application (also important for archiving purposes, see vi).

	1	2	3	4	5	
subitem not at all important	○	○	○	○	◉	essential

Clear selection

Does your paper address subitem 5-vii? *

Copy and paste relevant sections from the manuscript (include quotes in quotation marks "like this" to indicate direct quotes from your manuscript), or elaborate on this item by providing additional information not in the ms, or briefly explain why the item is not applicable/relevant for your study

"Upon completion, those interested in proceeding with the study were mailed a wifi scale (Fitbit Aria) and asked to provide staff with login info for the scale so that weight could be recorded for assessments. After randomization, participants had a 60-minute call with a study staff person to receive guidance on how to download and use their assigned app and be entered into their assigned Facebook group. Participants were allowed to keep the scale and were compensated for completing assessments."

5-viii) Mode of delivery, features/functionalities/components of the intervention and comparator, and the theoretical framework

Describe mode of delivery, features/functionalities/components of the intervention and comparator, and the theoretical framework [6] used to design them (instructional strategy [1], behaviour change techniques, persuasive features, etc., see e.g., [7, 8] for terminology). This includes an in-depth description of the content (including where it is coming from and who developed it) [1],” whether [and how] it is tailored to individual circumstances and allows users to track their progress and receive feedback” [6]. This also includes a description of communication delivery channels and – if computer-mediated communication is a component – whether communication was synchronous or asynchronous [6]. It also includes information on presentation strategies [1], including page design principles, average amount of text on pages, presence of hyperlinks to other resources, etc. [1].

	1	2	3	4	5	
subitem not at all important	○	○	○	○	◉	essential

Clear selection

Does your paper address subitem 5-viii? *

Copy and paste relevant sections from the manuscript (include quotes in quotation marks "like this" to indicate direct quotes from your manuscript), or elaborate on this item by providing additional information not in the ms, or briefly explain why the item is not applicable/relevant for your study

"Participants in both conditions were assigned one of the diet tracking apps and received the Diabetes Prevention Program (DPP) lifestyle intervention delivered within a counselor-led private Facebook group that included all participants in their respective condition. Participants randomized to the Slip Buddy condition were provided the Slip Buddy App, and participants randomized to the Calorie Tracking condition were instructed to install the free, commercially-available MyFitnessPal app. Each group had a different counselor who was either a registered dietitian or a clinical psychologist and each was trained in the app assigned to their respective conditions.
As in our previous work,[17, 18] the lifestyle intervention was delivered via twice daily posts and each week's content was based on the corresponding module of the DPP. The DPP assigns participants the goals of 1) calorie tracking to achieve a calorie goal based on amount needed to lose 1-2 pounds per week (modified to weekly slip tracking in the Slip Buddy condition), 2) developing a healthy diet consistent with the American Heart Association guidelines, 3) engaging in 150-300 minutes per week of moderate intensity exercise, 4) developing a strength training regimen consistent with the National Guidelines for Physical Activity, and 5) losing 1-2 pounds per week. Goal setting occurred on Monday mornings when the counselor asked participants to set 2-3 diet and exercise goals and gave specific suggestions based on the topic of the week (e.g., self-monitoring, reduce added sugar, add 15 minutes of exercise) to help participants progress toward their weight loss goal. On Fridays, the counselor put up a weigh-in post asking participants to reply with their weight change in pounds (e.g., lost 1 pound) for the week. This ensures participants are weighing themselves weekly and allows an opportunity for problem solving for those not losing weight. Goal accountability occurred every Sunday when the counselor asked participants to report how they did on their weekly goals. In between these key posts were posts related to the topic of the week (e.g., nutrition, making time for exercise). On remaining days the counselor posted discussion threads relating to that week's module. Each week staff produced weight and engagement reports for the counselors so they could identify participants who hadn't engaged in the past week and/or who were not losing weight and attempt to engage them in the group by tagging them in posts. Tagging a participant in a post results in their receiving a notification on their Facebook account that when clicked leads them to the post in which they were tagged. In our previous studies, this Facebook-delivered weight loss intervention [illegible]

has produced mean weight losses at 12 weeks ranging from 2.6%-4.8%.[19][20]
Slip Buddy App
As described above, we developed Slip Buddy App which assists users to track non-homeostatic eating and the contextual factors surrounding it (Figure 1).[16] Because "non-homeostatic eating" is scientific jargon, the app refers to these episodes as "slips." Participants were instructed to hit an "Oops!" button each time they have a diet slip, defined as any eating that resulted in consuming (food or drink) more than planned at a meal or between meals, eating in the absence of hunger (e.g., ate a donut someone brought to work), emotional eating, eating past the point of fullness, or an unhealthy food choice (e.g., stopped for fast food instead of cooking). The definition of a slip appeared near the "Oops!" button as a reminder to the user. To increase awareness of when slips are most likely to occur, the app passively collected the date and time for each slip reported. For each slip, participants were asked to rate their stress and hunger/satiety on 0-10 scales, to describe the context of the slip from drop-down menus, including type of eating episode (e.g., lunch, snack) and activity during the episode (e.g., working, socializing, watching TV). They were also asked to type in their location (e.g., restaurant), food consumed, and any other notable details they wanted to remember later in open text boxes. A check-in tab asks participants to report weight, hours slept last night, stress, and hunger in the morning and stress and hunger in the afternoon, but unlike in our first pilot study, we removed the notifications for the morning and afternoon check-ins to keep notifications to a minimum. The only notification occurred at the end of the day, asking participants if they missed entering any slips for the day and if so, to record the missed slips. The data collected by the app was securely sent to the remote Slip Buddy database server in addition to being recorded in the local database in participants' mobile phones. The history tab showed participants their past slip entries so they can look for patterns in contextual factors such as stress ratings, hunger level, activities, and/or location (see Figure 1). During the group each week, the counselor instructed participants to view their slip history from the previous week and use that information to set goals around how to avoid and/or manage cues associated with past slips. For example, if most slips occurred while watching TV in the evening, they could set the goals of planning healthy snacks at this time or reducing TV time. Participants were urged to use the app to learn when and why they slip and to reduce their slips over time toward the goal of losing 1-2 pounds per week. In the Facebook group, the DPP content relating to tracking and cues was modified to address slip tracking and to draw participants attention to the eating cues they were learning about from their Slip Buddy history. Participants randomized to the Calorie Tracking condition were instructed to download MyFitnessPal, a free, commercially-available mobile app that provides users a personalized calorie goal and allows them to track their caloric intake and energy

expenditure via exercise in an effort to stay within that goal. Participants were asked to enter everything they eat and drink throughout each day and all of their structured physical activity. They were asked to stay within their calorie goal to facilitate a weight loss of 1-2 pounds per week."

5-ix) Describe use parameters

Describe use parameters (e.g., intended "doses" and optimal timing for use). Clarify what instructions or recommendations were given to the user, e.g., regarding timing, frequency, heaviness of use, if any, or was the intervention used ad libitum.

	1	2	3	4	5	
subitem not at all important	○	○	○	○	◉	essential

Clear selection

Does your paper address subitem 5-ix?

Copy and paste relevant sections from the manuscript (include quotes in quotation marks "like this" to indicate direct quotes from your manuscript), or elaborate on this item by providing additional information not in the ms, or briefly explain why the item is not applicable/relevant for your study

"Participants were instructed to hit an “Oops!” button each time they have a diet slip, defined as any eating that resulted in consuming (food or drink) more than planned at a meal or between meals, eating in the absence of hunger (e.g., ate a donut someone brought to work), emotional eating, eating past the point of fullness, or an unhealthy food choice (e.g., stopped for fast food instead of cooking). The definition of a slip appeared near the “Oops!” button as a reminder to the user. To increase awareness of when slips are most likely to occur, the app passively collected the date and time for each slip reported. For each slip, participants were asked to rate their stress and hunger/satiety on 0-10 scales, to describe the context of the slip from drop-down menus, including type of eating episode (e.g., lunch, snack) and activity during the episode (e.g., working, socializing, watching TV). They were also asked to type in their location (e.g., restaurant), food consumed, and any other notable details they wanted to remember later in open text boxes. A check-in tab asks participants to report weight, hours slept last night, stress, and hunger in the morning and stress and hunger in the afternoon"

5-x) Clarify the level of human involvement

Clarify the level of human involvement (care providers or health professionals, also technical assistance) in the e-intervention or as co-intervention (detail number and expertise of professionals involved, if any, as well as “type of assistance offered, the timing and frequency of the support, how it is initiated, and the medium by which the assistance is delivered”. It may be necessary to distinguish between the level of human involvement required for the trial, and the level of human involvement required for a routine application outside of a RCT setting (discuss under item 21 – generalizability).

	1	2	3	4	5	
subitem not at all important	○	○	○	○	●	essential

Clear selection

Does your paper address subitem 5-x?

Copy and paste relevant sections from the manuscript (include quotes in quotation marks "like this" to indicate direct quotes from your manuscript), or elaborate on this item by providing additional information not in the ms, or briefly explain why the item is not applicable/relevant for your study

"Participants randomized to the Slip Buddy condition were provided the Slip Buddy App, and participants randomized to the Calorie Tracking condition were instructed to install the free, commercially-available MyFitnessPal app. Each group had a different counselor who was either a registered dietitian or a clinical psychologist and each was trained in the app assigned to their respective conditions.
As in our previous work,[17, 18] the lifestyle intervention was delivered via twice daily posts and each week's content was based on the corresponding module of the DPP. The DPP assigns participants the goals of 1) calorie tracking to achieve a calorie goal based on amount needed to lose 1-2 pounds per week (modified to weekly slip tracking in the Slip Buddy condition), 2) developing a healthy diet consistent with the American Heart Association guidelines, 3) engaging in 150-300 minutes per week of moderate intensity exercise, 4) developing a strength training regimen consistent with the National Guidelines for Physical Activity, and 5) losing 1-2 pounds per week. Goal setting occurred on Monday mornings when the counselor asked participants to set 2-3 diet and exercise goals and gave specific suggestions based on the topic of the week (e.g., self-monitoring, reduce added sugar, add 15 minutes of exercise) to help participants progress toward their weight loss goal. On Fridays, the counselor put up a weigh-in post asking participants to reply with their weight change in pounds (e.g., lost 1 pound) for the week. This ensures participants are weighing themselves weekly and allows an opportunity for problem solving for those not losing weight. Goal accountability occurred every Sunday when the counselor asked participants to report how they did on their weekly goals. In between these key posts were posts related to the topic of the week (e.g., nutrition, making time for exercise). On remaining days the counselor posted discussion threads relating to that week's module. Each week staff produced weight and engagement reports for the counselors so they could identify participants who hadn't engaged in the past week and/or who were not losing weight and attempt to engage them in the group by tagging them in posts. Tagging a participant in a post results in their receiving a notification on their Facebook account that when clicked leads them to the post in which they were tagged. In our previous studies, this Facebook-delivered weight loss intervention has produced mean weight losses at 12 weeks ranging from 2.6%-4.8%.[19][20]"

5-xi) Report any prompts/reminders used

Report any prompts/reminders used: Clarify if there were prompts (letters, emails, phone calls, SMS) to use the application, what triggered them, frequency etc. It may be necessary to distinguish between the level of prompts/reminders required for the trial, and the level of prompts/reminders for a routine application outside of a RCT setting (discuss under item 21 – generalizability).

	1	2	3	4	5	
subitem not at all important	○	○	○	○	◉	essential

Clear selection

Does your paper address subitem 5-xi? *

Copy and paste relevant sections from the manuscript (include quotes in quotation marks "like this" to indicate direct quotes from your manuscript), or elaborate on this item by providing additional information not in the ms, or briefly explain why the item is not applicable/relevant for your study

"Participants randomized to the Slip Buddy condition were provided the Slip Buddy App, and participants randomized to the Calorie Tracking condition were instructed to install the free, commercially-available MyFitnessPal app. Each group had a different counselor who was either a registered dietitian or a clinical psychologist and each was trained in the app assigned to their respective conditions and led the Facebook group for that condition.
As in our previous work,[17, 18] the lifestyle intervention was delivered via twice daily posts and each week's content was based on the corresponding module of the DPP. The DPP assigns participants the goals of 1) calorie tracking to achieve a calorie goal based on amount needed to lose 1-2 pounds per week (modified to weekly slip tracking in the Slip Buddy condition), 2) developing a healthy diet consistent with the American Heart Association guidelines, 3) engaging in 150-300 minutes per week of moderate intensity exercise, 4) developing a strength training regimen consistent with the National Guidelines for Physical Activity, and 5) losing 1-2 pounds per week. Goal setting occurred on Monday mornings when the counselor asked participants to set 2-3 diet and exercise goals and gave specific suggestions based on the topic of the week (e.g., self-monitoring, reduce added sugar, add 15 minutes of exercise) to help participants progress toward their weight loss goal. On Fridays, the counselor put up a weigh-in post asking participants to reply with their weight change in pounds (e.g., lost 1 pound) for the week. This ensures participants are weighing themselves weekly and allows an opportunity for problem solving for those not losing weight. Goal accountability occurred every Sunday when the counselor asked participants to report how they did on their weekly goals. In between these key posts were posts related to the topic of the week (e.g., nutrition, making time for exercise). On remaining days the counselor posted discussion threads relating to that week's module. Each week staff produced weight and engagement reports for the counselors so they could identify participants who hadn't engaged in the past week and/or who were not losing weight and attempt to engage them in the group by tagging them in posts. Tagging a participant in a post results in their receiving a notification on their Facebook account that when clicked leads them to the post in which they were tagged. In our previous studies, this Facebook-delivered weight loss intervention has produced mean weight losses at 12 weeks ranging from 2.6%-4.8%.[19][20]"

5-xii) Describe any co-interventions (incl. training/support)

Describe any co-interventions (incl. training/support): Clearly state any interventions that are provided in addition to the targeted eHealth intervention, as ehealth intervention may not be designed as stand-alone intervention. This includes training sessions and support [1]. It may be necessary to distinguish between the level of training required for the trial, and the level of training for a routine application outside of a RCT setting (discuss under item 21 – generalizability.

	1	2	3	4	5	
subitem not at all important	○	○	○	○	◉	essential

Clear selection

Does your paper address subitem 5-xii? *

Copy and paste relevant sections from the manuscript (include quotes in quotation marks "like this" to indicate direct quotes from your manuscript), or elaborate on this item by providing additional information not in the ms, or briefly explain why the item is not applicable/relevant for your study

"As in our previous work,[17, 18] the lifestyle intervention was delivered via twice daily posts and each week's content was based on the corresponding module of the DPP. The DPP assigns participants the goals of 1) calorie tracking to achieve a calorie goal based on amount needed to lose 1-2 pounds per week (modified to weekly slip tracking in the Slip Buddy condition), 2) developing a healthy diet consistent with the American Heart Association guidelines, 3) engaging in 150-300 minutes per week of moderate intensity exercise, 4) developing a strength training regimen consistent with the National Guidelines for Physical Activity, and 5) losing 1-2 pounds per week. Goal setting occurred on Monday mornings when the counselor asked participants to set 2-3 diet and exercise goals and gave specific suggestions based on the topic of the week (e.g., self-monitoring, reduce added sugar, add 15 minutes of exercise) to help participants progress toward their weight loss goal. On Fridays, the counselor put up a weigh-in post asking participants to reply with their weight change in pounds (e.g., lost 1 pound) for the week. This ensures participants are weighing themselves weekly and allows an opportunity for problem solving for those not losing weight. Goal accountability occurred every Sunday when the counselor asked participants to report how they did on their weekly goals. In between these key posts were posts related to the topic of the week (e.g., nutrition, making time for exercise). On remaining days the counselor posted discussion threads relating to that week's module. Each week staff produced weight and engagement reports for the counselors so they could identify participants who hadn't engaged in the past week and/or who were not losing weight and attempt to engage them in the group by tagging them in posts. Tagging a participant in a post results in their receiving a notification on their Facebook account that when clicked leads them to the post in which they were tagged. In our previous studies, this Facebook-delivered weight loss intervention has produced mean weight losses at 12 weeks ranging from 2.6%-4.8%.[19][20]"

6a) Completely defined pre-specified primary and secondary outcome measures, including how and when they were assessed

Does your paper address CONSORT subitem 6a? *

Copy and paste relevant sections from the manuscript (include quotes in quotation marks "like this" to indicate direct quotes from your manuscript), or elaborate on this item by providing additional information not in the ms, or briefly explain why the item is not applicable/relevant for your study

"Retention
Retention was defined as the percent in each condition completing the 12-week follow-up measures which included weight and a survey.
App Use
As the nature of diet tracking differed between the two treatment conditions, how we assessed app use also differed. For participants in the Slip Buddy condition, we intended to categorize participants as having used the Slip Buddy App for a given day if (1) backend data from the app revealed at least one slip was recorded or check-in completed (optional), or (2) in the absence of slips, the participants responded to the end-of-day check-in saying that they did not have any slips that day. Staff reviewed the data in the Slip Buddy database server (i.e., backend data) and recorded the number of days each week each participant used the app (e.g., either recorded a slip or responded to a notification or check-in reporting that they experienced no slips). However, some participants reported that they did not see or receive the end-of-day check-in notifications from the app which we determined was related to the authentication token on the phone expiring periodically. The end-of-the-day notification gives the participant the opportunity to confirm that no slips occurred if none had been recorded thus far. Without the notification we cannot distinguish between a day in which the participant did not track slips and a day in which no slips occurred. For this reason, backend data would be an underestimation of app use. Because failure can sometimes happen while transmitting app data to the remote database server, we also collected self-report app use data by emailing participants each week a single item asking them how many days they used the app to track slips that week. Self-report data was available for 74% (n=282/379) of weeks across all participants (counting only 7 weeks for the participant who withdrew due to pregnancy). Because 26% of self-report data was missing and backend data was incomplete by an unknown amount, we leveraged both forms of data to measure app use. We used the larger of the two values for two reasons: 1) when self-report data is higher than backend data it could correct for the underestimation bias of backend data, and 2) when backend data is higher than self-report it could correct for recall bias from self-report. The weakness is that we don’t have a way to correct for recall bias from self-report that overestimates use which surely exists to some extent. Self-report data were used on 58% of total weeks (220 weeks) and backend data were used 27% of weeks (104 weeks) which includes the 7 weeks backend was higher than [illegible]

self-report and the 97 weeks in which self-report was missing. On the remaining 15% of weeks (55 weeks) self-report and backend were the same so that value was used. Even though there is no way to correct for possible overestimations via self-report, on 26% of weeks (97 weeks) only backend data were available which would be an underestimate for those weeks.
For participants in the Calorie Tracking condition, research staff reviewed MyFitnessPal records and we coded a “complete day” of calorie tracking any day in which participants tracked 2+ meals and 800+ kcal/day, as has been done elsewhere.[21, 22] As participants in the Calorie Tracking condition were instructed to track all food and beverage intake, we only included “complete days” of tracking in our calculations of MyFitnessPal use.
Using the above definitions, we calculated the number and percentage of days participants in each treatment condition used their assigned app over the 12-week intervention. Because the Slip Buddy App was down for two days in week 3, participants in this condition could have only used the app on a maximum of 82 days versus the 84 possible days for participants in the Calorie Tracking condition. We also categorized participants in both conditions as to whether they used their assigned app at least once over the 12-week intervention, and whether they used their assigned app during week 12 as a measure of sustained engagement. Two participants were withdrawn or dropped out of the intervention due to incident pregnancy. For these women, use was not assessed after they were no longer in the intervention (after week 7 for the Slip Buddy participant who became pregnant, after week 2 for the Calorie Tracking participant), but instead the calculation of percent days the app was used only counted days they were in the intervention.
Slip Buddy App Data (Slip Buddy participants only)

Slips Reported
Backend data from the app was used to describe the number of slips reported for each participant during the intervention period and contextual factors related to slips.
Location of Slip
Participants were asked to note via free response where they were when the slip occurred. The first author collapsed free responses into categories that included work, home, other person’s house, restaurant/bar, at an event (e.g., football game), in the car, or at the gym.
Nature of Eating Episode
Participants also indicated the nature of the eating episode in which the slip occurred which included the choices breakfast, lunch, dinner, dessert, snack, or alcohol. Alcohol was included to capture drinking episodes that happen outside of the context of meals or snacks and to prompt participants to think of excess alcohol intake as a dietary slip.

Activity during Slip
Participants also indicated what they were doing from a drop-down menu of domestic activities (e.g., chores), working/studying (e.g., employment, school), socializing, screen time, or commuting.
Stress and Hunger/Fullness Ratings of Slips
When they entered a slip, participants rated how much stress they were experiencing before their slip on a 0 to 10 scale where 0 indicates no stress and 10 indicates extreme stress. Stress scores of 5 and above were considered moderate to high stress, whereas stress ratings of less than 5 were considered low stress. Participants also rated how hungry or full they felt before they slipped on a 0-10 scale where 0 indicates extremely hungry, 5 indicates comfortably full, and 10 indicates stuffed, i.e., uncomfortably full.
My Fitness Pal Data (Calorie Tracking participants only)
Participants were asked to record their diet every day for 12-weeks. This data was extracted from MyFitnessPal and coded for analysis. The first level of coding included recording each day that the participant entered at least one item. The second level was extracting the number of eating episodes and calories each day.
Usability
The System Usability Scale (SUS)[23] was used at 12-weeks to assess the usability of Slip Buddy App. The SUS is a 10-item 5-point Likert scale questionnaire regarding human-computer interaction. For one participant who only answered 9 of the 10 questions, we used their mean of those 9 items to impute a response to the tenth item. A SUS score above 70 is considered acceptable and above average while a score above 85 is considered excellent.[24] Moreover, when users rate a system with a SUS score of 82 (+-5), they tend to be “promoters” of the system, meaning likely to recommend it to a friend.[25]
Acceptability
At 12-weeks, participants in both conditions rated the helpfulness and ease of use of their assigned app (response options: strongly disagree, disagree, neutral, agree, strongly agree). We dichotomized responses as strongly agree/agree vs strongly disagree/disagree/neutral. Because Slip Buddy App is exclusively focused on diet, unlike calorie tracking apps which address both diet and exercise, we included a question at follow-up asking participants to rate whether a feature that would allow them to track exercise slips (i.e., times when they had planned to exercise but did not follow through) would increase the effectiveness of Slip Buddy App.
Acceptability was also evaluated in post-intervention focus groups via two questions, “what did you like most about Slip Buddy App and why?” and “what did you like least about Slip Buddy App and why?” During the intervention, participants started a discussion about the possibility of Slip Buddy App having a feature that would allow people to track when they were tempted to slip but resisted that temptation. Given the enthusiasm for the idea, we added a question to the focus

group script asking participants about the extent to which they would like to track temptations that did not turn into slips.
Burden
At 12-weeks, participants in both conditions rated how burdensome it was to use their assigned app on a scale of 0-100 with 0 being not at all burdensome and 100 being very burdensome. Participants rated how much they agreed that the app was time consuming, taxing, and tedious (response options: strongly disagree, disagree, neutral, agree, strongly agree). We dichotomized responses as strongly agree/agree vs strongly disagree/disagree/neutral.
Weight
Weight was obtained at baseline and 12 weeks from the wi-fi scales sent to participants upon enrollment."

6a-i) Online questionnaires: describe if they were validated for online use and apply CHERRIES items to describe how the questionnaires were designed/deployed

If outcomes were obtained through online questionnaires, describe if they were validated for online use and apply CHERRIES items to describe how the questionnaires were designed/deployed [9].

	1	2	3	4	5	
subitem not at all important	(●)	○	○	○	○	essential

Clear selection

Does your paper address subitem 6a-i?

Copy and paste relevant sections from manuscript text

Your answer

6a-ii) Describe whether and how “use” (including intensity of use/dosage) was defined/measured/monitored

Describe whether and how “use” (including intensity of use/dosage) was defined/measured/monitored (logins, logfile analysis, etc.). Use/adoption metrics are important process outcomes that should be reported in any ehealth trial.

	1	2	3	4	5	
subitem not at all important	○	○	○	○	◉	essential

Clear selection

Does your paper address subitem 6a-ii?

Copy and paste relevant sections from manuscript text

As the nature of diet tracking differed between the two treatment conditions, how we assessed app use also differed. For participants in the Slip Buddy condition, we intended to categorize participants as having used the Slip Buddy App for a given day if (1) backend data from the app revealed at least one slip was recorded or check-in completed (optional), or (2) in the absence of slips, the participants responded to the end-of-day check-in saying that they did not have any slips that day. Staff reviewed the data in the Slip Buddy database server (i.e., backend data) and recorded the number of days each week each participant used the app (e.g., either recorded a slip or responded to a notification or check-in reporting that they experienced no slips). However, some participants reported that they did not see or receive the end-of-day check-in notifications from the app which we determined was related to the authentication token on the phone expiring periodically. The end-of-the-day notification gives the participant the opportunity to confirm that no slips occurred if none had been recorded thus far. Without the notification we cannot distinguish between a day in which the participant did not track slips and a day in which no slips occurred. For this reason, backend data would be an underestimation of app use. Because failure can sometimes happen while transmitting app data to the remote database server, we also collected self-report app use data by emailing participants each week a single item asking them how many days they used the app to track slips that week. Self-report data was available for 74% (n=282/379) of weeks across all participants (counting only 7 weeks for the participant who withdrew due to pregnancy). Because 26% of self-report data was missing and backend data was incomplete by an unknown amount, we leveraged both forms of data to measure app use. We used the larger of the two values for two reasons: 1) when self-report data is higher than backend data it could correct for the underestimation bias of backend data, and 2) when backend data is higher than self-report it could correct for recall bias from self-report. The weakness is that we don’t have a way to correct for recall bias from self-report that overestimates use which surely exists to some extent. Self-report data were used on 58% of total weeks (220 weeks) and backend data were used 27% of weeks (104 weeks) which includes the 7 weeks backend was higher than self-report and the 97 weeks in which self-report was missing. On the remaining 15% of weeks (55 weeks) self-report and backend were the same so that value was used. Even though there is no way to correct for possible overestimations via self-report, on 26% of weeks (97 weeks) only backend data were available which would be an underestimate for those weeks.

For participants in the Calorie Tracking condition, research staff reviewed

[illegible]
MyFitnessPal records and we coded a “complete day” of calorie tracking any day in which participants tracked 2+ meals and 800+ kcal/day, as has been done elsewhere.[21, 22] As participants in the Calorie Tracking condition were instructed to track all food and beverage intake, we only included “complete days” of tracking in our calculations of MyFitnessPal use.
Using the above definitions, we calculated the number and percentage of days participants in each treatment condition used their assigned app over the 12-week intervention. Because the Slip Buddy App was down for two days in week 3, participants in this condition could have only used the app on a maximum of 82 days versus the 84 possible days for participants in the Calorie Tracking condition. We also categorized participants in both conditions as to whether they used their assigned app at least once over the 12-week intervention, and whether they used their assigned app during week 12 as a measure of sustained engagement. Two participants were withdrawn or dropped out of the intervention due to incident pregnancy. For these women, use was not assessed after they were no longer in the intervention (after week 7 for the Slip Buddy participant who became pregnant, after week 2 for the Calorie Tracking participant), but instead the calculation of percent days the app was used only counted days they were in the intervention.

6a-iii) Describe whether, how, and when qualitative feedback from participants was obtained

Describe whether, how, and when qualitative feedback from participants was obtained (e.g., through emails, feedback forms, interviews, focus groups).

	1	2	3	4	5	
subitem not at all important	○	○	○	○	◉	essential

Clear selection

Does your paper address subitem 6a-iii?

Copy and paste relevant sections from manuscript text

"Acceptability was also evaluated in post-intervention focus groups via two questions, “what did you like most about Slip Buddy App and why?” and “what did you like least about Slip Buddy App and why?” During the intervention, participants started a discussion about the possibility of Slip Buddy App having a feature that would allow people to track when they were tempted to slip but resisted that temptation. Given the enthusiasm for the idea, we added a question to the focus group script asking participants about the extent to which they would like to track temptations that did not turn into slips."

6b) Any changes to trial outcomes after the trial commenced, with reasons

Does your paper address CONSORT subitem 6b? *

Copy and paste relevant sections from the manuscript (include quotes in quotation marks "like this" to indicate direct quotes from your manuscript), or elaborate on this item by providing additional information not in the ms, or briefly explain why the item is not applicable/relevant for your study

NA

7a) How sample size was determined

NPT: When applicable, details of whether and how the clustering by care provides or centers was addressed

7a-i) Describe whether and how expected attrition was taken into account when calculating the sample size

Describe whether and how expected attrition was taken into account when calculating the sample size.

	1	2	3	4	5	
subitem not at all important	(●)	( )	( )	( )	( )	essential

Clear selection

Does your paper address subitem 7a-i?

Copy and paste relevant sections from manuscript title (include quotes in quotation marks "like this" to indicate direct quotes from your manuscript), or elaborate on this item by providing additional information not in the ms, or briefly explain why the item is not applicable/relevant for your study

NA a feasibility trial

7b) When applicable, explanation of any interim analyses and stopping guidelines

Does your paper address CONSORT subitem 7b? *

Copy and paste relevant sections from the manuscript (include quotes in quotation marks "like this" to indicate direct quotes from your manuscript), or elaborate on this item by providing additional information not in the ms, or briefly explain why the item is not applicable/relevant for your study

NA

8a) Method used to generate the random allocation sequence

NPT: When applicable, how care providers were allocated to each trial group

Does your paper address CONSORT subitem 8a? *

Copy and paste relevant sections from the manuscript (include quotes in quotation marks "like this" to indicate direct quotes from your manuscript), or elaborate on this item by providing additional information not in the ms, or briefly explain why the item is not applicable/relevant for your study

Participants were randomized 1:1 into the two conditions

8b) Type of randomisation; details of any restriction (such as blocking and block size)

Does your paper address CONSORT subitem 8b? *

Copy and paste relevant sections from the manuscript (include quotes in quotation marks "like this" to indicate direct quotes from your manuscript), or elaborate on this item by providing additional information not in the ms, or briefly explain why the item is not applicable/relevant for your study

no

9) Mechanism used to implement the random allocation sequence (such as sequentially numbered containers), describing any steps taken to conceal the sequence until interventions were assigned

Does your paper address CONSORT subitem 9? *

Copy and paste relevant sections from the manuscript (include quotes in quotation marks "like this" to indicate direct quotes from your manuscript), or elaborate on this item by providing additional information not in the ms, or briefly explain why the item is not applicable/relevant for your study

SAS

10) Who generated the random allocation sequence, who enrolled participants, and who assigned participants to interventions

Does your paper address CONSORT subitem 10? *

Copy and paste relevant sections from the manuscript (include quotes in quotation marks "like this" to indicate direct quotes from your manuscript), or elaborate on this item by providing additional information not in the ms, or briefly explain why the item is not applicable/relevant for your study

Molly Waring

11a) If done, who was blinded after assignment to interventions (for example, participants, care providers, those assessing outcomes) and how

NPT: Whether or not administering co-interventions were blinded to group assignment

11a-i) Specify who was blinded, and who wasn't

Specify who was blinded, and who wasn't. Usually, in web-based trials it is not possible to blind the participants [1, 3] (this should be clearly acknowledged), but it may be possible to blind outcome assessors, those doing data analysis or those administering co-interventions (if any).

	1	2	3	4	5	
subitem not at all important	(●)	( )	( )	( )	( )	essential

Clear selection

Does your paper address subitem 11a-i? *

Copy and paste relevant sections from the manuscript (include quotes in quotation marks "like this" to indicate direct quotes from your manuscript), or elaborate on this item by providing additional information not in the ms, or briefly explain why the item is not applicable/relevant for your study

blinding was not possible

11a-ii) Discuss e.g., whether participants knew which intervention was the "intervention of interest" and which one was the "comparator"

Informed consent procedures (4a-ii) can create biases and certain expectations - discuss e.g., whether participants knew which intervention was the "intervention of interest" and which one was the "comparator".

	1	2	3	4	5	
subitem not at all important	○	○	◉	○	○	essential

Clear selection

Does your paper address subitem 11a-ii?

Copy and paste relevant sections from the manuscript (include quotes in quotation marks "like this" to indicate direct quotes from your manuscript), or elaborate on this item by providing additional information not in the ms, or briefly explain why the item is not applicable/relevant for your study

Participants were told we were testing two different forms of diet tracking.

11b) If relevant, description of the similarity of interventions

(this item is usually not relevant for ehealth trials as it refers to similarity of a placebo or sham intervention to a active medication/intervention)

Does your paper address CONSORT subitem 11b? *

Copy and paste relevant sections from the manuscript (include quotes in quotation marks "like this" to indicate direct quotes from your manuscript), or elaborate on this item by providing additional information not in the ms, or briefly explain why the item is not applicable/relevant for your study

"Participants in both conditions were assigned one of the diet tracking apps and received the Diabetes Prevention Program (DPP) lifestyle intervention delivered within a counselor-led private Facebook group that included all participants in their respective condition. Participants randomized to the Slip Buddy condition were provided the Slip Buddy App, and participants randomized to the Calorie Tracking condition were instructed to install the free, commercially-available MyFitnessPal app. Each group had a different counselor who was either a registered dietitian or a clinical psychologist and each was trained in the app assigned to their respective conditions and led the Facebook group for that condition.
As in our previous work,[17, 18] the lifestyle intervention was delivered via twice daily posts and each week's content was based on the corresponding module of the DPP. The DPP assigns participants the goals of 1) calorie tracking to achieve a calorie goal based on amount needed to lose 1-2 pounds per week (modified to weekly slip tracking in the Slip Buddy condition), 2) developing a healthy diet consistent with the American Heart Association guidelines, 3) engaging in 150-300 minutes per week of moderate intensity exercise, 4) developing a strength training regimen consistent with the National Guidelines for Physical Activity, and 5) losing 1-2 pounds per week. Goal setting occurred on Monday mornings when the counselor asked participants to set 2-3 diet and exercise goals and gave specific suggestions based on the topic of the week (e.g., self-monitoring, reduce added sugar, add 15 minutes of exercise) to help participants progress toward their weight loss goal. On Fridays, the counselor put up a weigh-in post asking participants to reply with their weight change in pounds (e.g., lost 1 pound) for the week. This ensures participants are weighing themselves weekly and allows an opportunity for problem solving for those not losing weight. Goal accountability occurred every Sunday when the counselor asked participants to report how they did on their weekly goals. In between these key posts were posts related to the topic of the week (e.g., nutrition, making time for exercise). On remaining days the counselor posted discussion threads relating to that week's module. Each week staff produced weight and engagement reports for the counselors so they could identify participants who hadn't engaged in the past week and/or who were not losing weight and attempt to engage them in the group by tagging them in posts. Tagging a participant in a post results in their receiving a notification on their Facebook account that when clicked leads them to the post in which they were tagged. In our previous studies, this Facebook-delivered weight loss intervention has produced mean weight losses at 12 weeks ranging from 2.6%-4.8%.[19][20]"

12a) Statistical methods used to compare groups for primary and secondary outcomes

NPT: When applicable, details of whether and how the clustering by care providers or centers was addressed

Does your paper address CONSORT subitem 12a? *

Copy and paste relevant sections from the manuscript (include quotes in quotation marks "like this" to indicate direct quotes from your manuscript), or elaborate on this item by providing additional information not in the ms, or briefly explain why the item is not applicable/relevant for your study

"We summarized retention, app use, slips, usability, acceptability, and burden using descriptive statistics. For variables that were normally distributed, we described distributions using mean and standard deviation (M[SD]), and for variables that were not normally distributed, we described distributions using median and inter-quartile range (IQR). We compared use, retention, usability, and acceptability by treatment condition using t-tests, chi-squared tests, or Fisher's exact tests as appropriate. We compared treatment conditions on app burden using a Wilcoxon rank-sum test. Insufficient retention, acceptability, and use was assumed if the calorie tracking condition showed a statistically significant advantage relative to Slip Buddy. Statistical tests were not used to compare groups on weight loss because this pilot study was not powered for weight loss efficacy thus it is not appropriate to perform such tests, as discussed elsewhere.[27] We took an intent-to-treat approach to describing weight change. Two participants (one in each condition) became pregnant during the study. We used latest available pre-pregnancy weights (from weeks 2 and 3, respectively) from their study scales as their follow-up values. Three participants did not provide weight at follow-up. We also used their latest weight from their study scales (weeks 6, 9, and 10, respectively) as their follow-up values. We secondarily report weight loss assuming no weight loss for the 2 participants who became pregnant and the 3 participants lost to follow-up (i.e., baseline observation carried forward), We secondarily reported weight change excluding these five participants for whom we do not have non-pregnant follow-up weight. We conducted a directed content analysis[26] of focus group data on acceptability. The first author developed a codebook based on themes emerging from participant responses. Two coders independently coded responses and discussion was used to achieve consensus on disagreements. Inter-rater reliability was also calculated.[28] We summarized the frequency of themes. Data management and quantitative analyses were conducted in SAS 9.4 (SAS Institute, Inc., Cary, NC). "

12a-i) Imputation techniques to deal with attrition / missing values

Imputation techniques to deal with attrition / missing values: Not all participants will use the intervention/comparator as intended and attrition is typically high in ehealth trials. Specify how participants who did not use the application or dropped out from the trial were treated in the statistical analysis (a complete case analysis is strongly discouraged, and simple imputation techniques such as LOCF may also be problematic [4]).

	1	2	3	4	5	
subitem not at all important	○	○	○	○	◉	essential

Clear selection

Does your paper address subitem 12a-i? *

Copy and paste relevant sections from the manuscript (include quotes in quotation marks "like this" to indicate direct quotes from your manuscript), or elaborate on this item by providing additional information not in the ms, or briefly explain why the item is not applicable/relevant for your study

"We took an intent-to-treat approach to describing weight change. Two participants (one in each condition) became pregnant during the study. We used latest available pre-pregnancy weights (from weeks 2 and 3, respectively) from their study scales as their follow-up values. Three participants did not provide weight at follow-up. We also used their latest weight from their study scales (weeks 6, 9, and 10, respectively) as their follow-up values. We secondarily report weight loss assuming no weight loss for the 2 participants who became pregnant and the 3 participants lost to follow-up (i.e., baseline observation carried forward), We secondarily reported weight change excluding these five participants for whom we do not have non-pregnant follow-up weight."

12b) Methods for additional analyses, such as subgroup analyses and adjusted analyses

Does your paper address CONSORT subitem 12b? *

Copy and paste relevant sections from the manuscript (include quotes in quotation marks "like this" to indicate direct quotes from your manuscript), or elaborate on this item by providing additional information not in the ms, or briefly explain why the item is not applicable/relevant for your study

NA

X26) REB/IRB Approval and Ethical Considerations [recommended as subheading under "Methods"] (not a CONSORT item)

X26-i) Comment on ethics committee approval

	1	2	3	4	5	
subitem not at all important	○	○	○	○	◉	essential

Clear selection

Does your paper address subitem X26-i?

Copy and paste relevant sections from the manuscript (include quotes in quotation marks "like this" to indicate direct quotes from your manuscript), or elaborate on this item by providing additional information not in the ms, or briefly explain why the item is not applicable/relevant for your study

"All work was approved by the University of Connecticut Institutional Review Board."

x26-ii) Outline informed consent procedures

Outline informed consent procedures e.g., if consent was obtained offline or online (how? Checkbox, etc.?), and what information was provided (see 4a-ii). See [6] for some items to be included in informed consent documents.

	1	2	3	4	5	
subitem not at all important	○	○	○	○	◉	essential

Clear selection

Does your paper address subitem X26-ii?

Copy and paste relevant sections from the manuscript (include quotes in quotation marks "like this" to indicate direct quotes from your manuscript), or elaborate on this item by providing additional information not in the ms, or briefly explain why the item is not applicable/relevant for your study

"Recruitment ads contained a link to a screening survey that included a study description, initial informed consent, and screening questions. Participants eligible after the screening survey were emailed the consent form and completed a telephone screening call. The screening call included a review of the consent form, any remaining eligibility-related questions, and an emailed link to the baseline survey."

X26-iii) Safety and security procedures

Safety and security procedures, incl. privacy considerations, and any steps taken to reduce the likelihood or detection of harm (e.g., education and training, availability of a hotline)

	1	2	3	4	5	
subitem not at all important	○	○	○	○	◉	essential

Clear selection

Does your paper address subitem X26-iii?

Copy and paste relevant sections from the manuscript (include quotes in quotation marks "like this" to indicate direct quotes from your manuscript), or elaborate on this item by providing additional information not in the ms, or briefly explain why the item is not applicable/relevant for your study

"After randomization, participants had a 60-minute call with a study staff person to receive guidance on how to download and use their assigned app and be entered into their assigned Facebook group." Privacy settings of Facebook were discussed on this call

RESULTS

13a) For each group, the numbers of participants who were randomly assigned, received intended treatment, and were analysed for the primary outcome

NPT: The number of care providers or centers performing the intervention in each group and the number of patients treated by each care provider in each center

Does your paper address CONSORT subitem 13a? *

Copy and paste relevant sections from the manuscript (include quotes in quotation marks "like this" to indicate direct quotes from your manuscript), or elaborate on this item by providing additional information not in the ms, or briefly explain why the item is not applicable/relevant for your study

see CONSORT diagram

13b) For each group, losses and exclusions after randomisation, together with reasons

Does your paper address CONSORT subitem 13b? (NOTE: Preferably, this is shown in a CONSORT flow diagram) *

Copy and paste relevant sections from the manuscript (include quotes in quotation marks "like this" to indicate direct quotes from your manuscript), or elaborate on this item by providing additional information not in the ms, or briefly explain why the item is not applicable/relevant for your study

CONSORT diagram in the paper illustrates this

13b-i) Attrition diagram

Strongly recommended: An attrition diagram (e.g., proportion of participants still logging in or using the intervention/comparator in each group plotted over time, similar to a survival curve) or other figures or tables demonstrating usage/dose/engagement.

	1	2	3	4	5	
subitem not at all important	○	○	○	○	◉	essential

Clear selection

Does your paper address subitem 13b-i?

Copy and paste relevant sections from the manuscript or cite the figure number if applicable (include quotes in quotation marks "like this" to indicate direct quotes from your manuscript), or elaborate on this item by providing additional information not in the ms, or briefly explain why the item is not applicable/relevant for your study

Figure 3

14a) Dates defining the periods of recruitment and follow-up

Does your paper address CONSORT subitem 14a? *

Copy and paste relevant sections from the manuscript (include quotes in quotation marks "like this" to indicate direct quotes from your manuscript), or elaborate on this item by providing additional information not in the ms, or briefly explain why the item is not applicable/relevant for your study

We conducted a pilot feasibility randomized trial in which participants who are overweight or obese were recruited into a remotely delivered intervention via online advertisements at the University of Connecticut, on ResearchMatch, and in Facebook groups across the US between July and October of 2019.

14a-i) Indicate if critical “secular events” fell into the study period

Indicate if critical “secular events” fell into the study period, e.g., significant changes in Internet resources available or “changes in computer hardware or Internet delivery resources”

	1	2	3	4	5	
subitem not at all important	(•)	( )	( )	( )	( )	essential

Clear selection

Does your paper address subitem 14a-i?

Copy and paste relevant sections from the manuscript (include quotes in quotation marks "like this" to indicate direct quotes from your manuscript), or elaborate on this item by providing additional information not in the ms, or briefly explain why the item is not applicable/relevant for your study

NA

14b) Why the trial ended or was stopped (early)

Does your paper address CONSORT subitem 14b? *

Copy and paste relevant sections from the manuscript (include quotes in quotation marks "like this" to indicate direct quotes from your manuscript), or elaborate on this item by providing additional information not in the ms, or briefly explain why the item is not applicable/relevant for your study

NA

15) A table showing baseline demographic and clinical characteristics for each group

NPT: When applicable, a description of care providers (case volume, qualification, expertise, etc.) and centers (volume) in each group

Does your paper address CONSORT subitem 15? *

Copy and paste relevant sections from the manuscript (include quotes in quotation marks "like this" to indicate direct quotes from your manuscript), or elaborate on this item by providing additional information not in the ms, or briefly explain why the item is not applicable/relevant for your study

Table 1

15-i) Report demographics associated with digital divide issues

In ehealth trials it is particularly important to report demographics associated with digital divide issues, such as age, education, gender, social-economic status, computer/Internet/ehealth literacy of the participants, if known.

	1	2	3	4	5	
subitem not at all important	○	○	○	○	◉	essential

Clear selection

Does your paper address subitem 15-i? *

Copy and paste relevant sections from the manuscript (include quotes in quotation marks "like this" to indicate direct quotes from your manuscript), or elaborate on this item by providing additional information not in the ms, or briefly explain why the item is not applicable/relevant for your study

Table 1

16) For each group, number of participants (denominator) included in each analysis and whether the analysis was by original assigned groups

16-i) Report multiple “denominators” and provide definitions

Report multiple “denominators” and provide definitions: Report N’s (and effect sizes) “across a range of study participation [and use] thresholds” [1], e.g., N exposed, N consented, N used more than x times, N used more than y weeks, N participants “used” the intervention/comparator at specific pre-defined time points of interest (in absolute and relative numbers per group). Always clearly define “use” of the intervention.

	1	2	3	4	5	
subitem not at all important	○	○	○	○	●	essential

Clear selection

Does your paper address subitem 16-i? *

Copy and paste relevant sections from the manuscript (include quotes in quotation marks "like this" to indicate direct quotes from your manuscript), or elaborate on this item by providing additional information not in the ms, or briefly explain why the item is not applicable/relevant for your study

Retention
Retention was high in both treatment conditions with 97% of Slip Buddy participants (n= 31) and 94% of Calorie Tracking participants (n=30) providing follow-up data (p=1.0000; Fisher's exact test).
App Use
Nearly all participants randomized to the Slip Buddy condition (97%; n=31/32) and the Calorie Tracking condition (97%; n=31/32) used their assigned app at least once during the 12-week intervention. Participants in the Slip Buddy condition used their assigned app on a mean of 44.0 (SD: 25.8) days and participants in the Calorie Tracking condition used their app on a mean of 46.3 days (SD: 24.0), which represented use on an average of 53.8% (SD: 31.3%) of possible days for participants in the Slip Buddy condition and 57.5% (SD: 28.4%) of possible days for participants in the Calorie Tracking condition [t(62)=.495, p=.44]. In terms of sustained use of their assigned app, 55% of Slip Buddy participants (n=18) used the app at least once in week 12 of the intervention compared to only 35% (n=11) of Calorie Tracking participants [X2 (1,N=62)=2.35; p=0.13]. The proportion of participants using their assigned app each week of the 12-week intervention period is shown in Figure 3.
Slips Reported
One participant did not use the Slip Buddy App and one participant responded to the notification but did not record any slips. The remaining participants reported a total of 538 slips during the 12-week intervention. Participants who reported slips (n=30) reported a median of 15 slips (inter-quartile range: 8-23; range: 2-66; M[SD]: 17.9 [14.4]). The majority of slips happened at home (n=297; 55.2%), followed by work (n=113; 21.0%), restaurant/bar (n=86; 16.0%; Table 2). The nature of the eating episode most likely to be reported as a slip was a snack (n=220; 40.9%), followed by dinner (n=102; 19.0%), dessert (n=77; 14.3%), and lunch (n=60; 11.2%; Table 2). Activities engaged in when the slip occurred was split over work or studying (n=153; 28.4%), socializing (n=131; 24.2%), screen time (n=123; 22.9%), and domestic activities (n=112; 20.8%); a small percent of slips occurred while commuting (n=20; 3.7%; Table 2). The median stress rating during slips was 4 (inter-quartile range: 2-5). The median hunger/fullness rating during slips was 4 (inter-quartile range: 2-5). One-fifth of slips (n=106; 20%) occurred when both stress and hunger were low, and another 20% (n=105) occurred when both stress and hunger were high.

Usability
Mean SUS score for the Slip Buddy condition was 64.8 (SD: 16.5) which is the marginally acceptable range. Comparatively, the mean SUS score for participants in the Calorie Tracking condition was 76.3 (SD: 17.6) which is considered good acceptability. Participants in the Calorie Tracking condition rated the MyFitnessPal app as more usable on average than Slip Buddy participants rated the Slip Buddy App (t(59)=2.64, p=0.0106). Usability issues reported during the intervention included the Slip Buddy App crashing on some phone models and a temporary outage, both of which were fixed during the study.
Acceptability
Among participants who completed the follow-up survey, 39% (n=12) of Slip Buddy participants agreed or strongly agreed that tracking slips was helpful, whereas 77% (n=23) of Calorie Tracking participants agreed or strongly agreed that tracking diet and exercise was helpful (X2 (1,N=61)=8.8916; p=0.0027; Table 3).

The majority of both Slip Buddy (n=24; 77%) and Calorie Tracking participants (n=26; 87%) agreed or strongly agreed that using their respective app was easy (X2 (1,N=61)=0.8820; p=0.3674; Table 3). Two-thirds of Slip Buddy participants (n=21; 68%) agreed or strongly agreed that adding the ability to track exercise slips would be helpful.
Burden
On a scale of 0 to 100, median burden rating for participants in the Slip Buddy condition was 30 (IQR: 15-50) and the median burden rating for participants in the Calorie Tracking condition was 45 (IQR: 10-60; U=949.5000, p=0.7829). The proportion of participants who agreed or strongly agreed that using their assigned app was tedious or taxing did not differ by treatment condition (Table 3). Finally, 20% (n=6) Slip Buddy participants agreed or strongly agreed that using Slip Buddy App was time consuming, while 43% (n=13) of MyFitnessPal participants agreed or strongly agreed that using MyFitnessPal was time consuming (X2 (1,N=61)=3.7741; p=0.0521; Table 2).
Feedback from participants in the Slip Buddy condition
Twenty-eight (88%) of participants in the Slip Buddy condition attended post-intervention focus groups and they made 35 responses to the question about what they liked most about Slip Buddy App (IRR=94%; Kappa=.89). The most common theme of responses was ease of use/simple concept (n=23; 66%), followed by increasing accountability and/or awareness of overeating and/or triggers (n=8; 23%), the end-of-day reminder to track slips (n=2; 5%), feeling motivated to not slip so there would be nothing to track (n=1; 3%) and other (n=1; 3%). Participants made 35 responses about what they liked least about Slip Buddy App (IRR=91%; Kappa=.89). Responses reflected the following themes: technical issues (e.g., app crashing, notifications not going away; n=10; 29%), easy to forget to use or not sure

how to use when no slips (n=7; 21%), did not find stress ratings relevant (n=5; 15%), focus on slips is too negative (n=4; 12%), was not sure what to count as a slip (n=4; 12%), did not include diet instruction (n=4; 12%), slip history screen was not as informative as it could be (n=1; 3%). For the final question regarding their thoughts on a feature that would allow them to track when they were tempted but did not slip, 75% (n=21) said they would be enthusiastic about this feature. The remainder said they worried it would add too much burden.

Weight Change

Over 12 weeks, participants randomized to the Slip Buddy condition had average weight loss of -6.5 pounds (SD=9.7) or 3.0% (SD: 4.5%) of their baseline weight and participants randomized to the Calorie Tracking condition had weight loss of -7.5 pounds (SD=10.7) or 3.6% (SD: 4.9%) of their baseline weight (Table 4). In terms of clinically significant weight loss, 31% and 34% of participants randomized to the Slip Buddy and Calorie Tracking conditions, respectively, achieved 5% or greater weight loss, and 47% and 47%, respectively, achieved 3% or greater weight loss (Table 4). In a secondary analysis assuming no weight loss for the 2 participants who became pregnant and the 3 participants lost to follow-up (i.e., baseline observation carried forward approach), weight losses were on average -6.1 (SD: 9.8) pounds and -2.8% (SD: 4.6%) among Slip Buddy participants and on average -7.1 (SD: 10.8) pounds and -3.4% (SD: 5.0%) among Calorie Tracking participants, with 31% and 34% of participants, respectively, losing 5% or greater weight loss from baseline, and 48% and 47%, respectively, losing 3% or greater weight loss from baseline. In a secondary analysis of just the 59 participants who provided weight at follow-up and were not pregnant (n=30 Slip Buddy, n=29 Calorie Tracking), weight losses were on average -6.5 (SD: 10.0) pounds and -3.0% (SD: 4.7%) among Slip Buddy participants and on average -7.9 (SD: 11.1) pounds and -3.8% (SD: 5.1%) among Calorie Tracking participants, with 33% (n=11) and 38% (n=12) of participants, respectively, losing 5% or greater weight loss from baseline and 44% (n=14) and 44% (n=14), respectively, losing 3% or greater weight loss.

16-ii) Primary analysis should be intent-to-treat

Primary analysis should be intent-to-treat, secondary analyses could include comparing only “users”, with the appropriate caveats that this is no longer a randomized sample (see 18-i).

	1	2	3	4	5	
subitem not at all important	○	○	○	○	◉	essential

Clear selection

Does your paper address subitem 16-ii?

Copy and paste relevant sections from the manuscript (include quotes in quotation marks "like this" to indicate direct quotes from your manuscript), or elaborate on this item by providing additional information not in the ms, or briefly explain why the item is not applicable/relevant for your study

We took an intent-to-treat approach to describing weight change.

17a) For each primary and secondary outcome, results for each group, and the estimated effect size and its precision (such as 95% confidence interval)

Does your paper address CONSORT subitem 17a? *

Copy and paste relevant sections from the manuscript (include quotes in quotation marks "like this" to indicate direct quotes from your manuscript), or elaborate on this item by providing additional information not in the ms, or briefly explain why the item is not applicable/relevant for your study

NA

17a-i) Presentation of process outcomes such as metrics of use and intensity of use

In addition to primary/secondary (clinical) outcomes, the presentation of process outcomes such as metrics of use and intensity of use (dose, exposure) and their operational definitions is critical. This does not only refer to metrics of attrition (13-b) (often a binary variable), but also to more continuous exposure metrics such as “average session length”. These must be accompanied by a technical description how a metric like a “session” is defined (e.g., timeout after idle time) [1] (report under item 6a).

	1	2	3	4	5	
subitem not at all important	○	○	○	○	◉	essential

Clear selection

Does your paper address subitem 17a-i?

Copy and paste relevant sections from the manuscript (include quotes in quotation marks "like this" to indicate direct quotes from your manuscript), or elaborate on this item by providing additional information not in the ms, or briefly explain why the item is not applicable/relevant for your study

Nearly all participants randomized to the Slip Buddy condition (97%; n=31/32) and the Calorie Tracking condition (97%; n=31/32) used their assigned app at least once during the 12-week intervention. Participants in the Slip Buddy condition used their assigned app on a mean of 44.0 (SD: 25.8) days and participants in the Calorie Tracking condition used their app on a mean of 46.3 days (SD: 24.0), which represented use on an average of 53.8% (SD: 31.3%) of possible days for participants in the Slip Buddy condition and 57.5% (SD: 28.4%) of possible days for participants in the Calorie Tracking condition [t(62)=.495, p=.44]. In terms of sustained use of their assigned app, 55% of Slip Buddy participants (n=18) used the app at least once in week 12 of the intervention compared to only 35% (n=11) of Calorie Tracking participants [X2 (1,N=62)=2.35; p=0.13]. The proportion of participants using their assigned app each week of the 12-week intervention period is shown in Figure 3.

17b) For binary outcomes, presentation of both absolute and relative effect sizes is recommended

Does your paper address CONSORT subitem 17b? *

Copy and paste relevant sections from the manuscript (include quotes in quotation marks "like this" to indicate direct quotes from your manuscript), or elaborate on this item by providing additional information not in the ms, or briefly explain why the item is not applicable/relevant for your study

NA

18) Results of any other analyses performed, including subgroup analyses and adjusted analyses, distinguishing pre-specified from exploratory

Does your paper address CONSORT subitem 18? *

Copy and paste relevant sections from the manuscript (include quotes in quotation marks "like this" to indicate direct quotes from your manuscript), or elaborate on this item by providing additional information not in the ms, or briefly explain why the item is not applicable/relevant for your study

NA

18-i) Subgroup analysis of comparing only users

A subgroup analysis of comparing only users is not uncommon in ehealth trials, but if done, it must be stressed that this is a self-selected sample and no longer an unbiased sample from a randomized trial (see 16-iii).

	1	2	3	4	5	
subitem not at all important	(●)	( )	( )	( )	( )	essential

Clear selection

Does your paper address subitem 18-i?

Copy and paste relevant sections from the manuscript (include quotes in quotation marks "like this" to indicate direct quotes from your manuscript), or elaborate on this item by providing additional information not in the ms, or briefly explain why the item is not applicable/relevant for your study

NA

19) All important harms or unintended effects in each group

(for specific guidance see CONSORT for harms)

Does your paper address CONSORT subitem 19? *

Copy and paste relevant sections from the manuscript (include quotes in quotation marks "like this" to indicate direct quotes from your manuscript), or elaborate on this item by providing additional information not in the ms, or briefly explain why the item is not applicable/relevant for your study

NA

19-i) Include privacy breaches, technical problems

Include privacy breaches, technical problems. This does not only include physical “harm” to participants, but also incidents such as perceived or real privacy breaches [1], technical problems, and other unexpected/unintended incidents. “Unintended effects” also includes unintended positive effects [2].

	1	2	3	4	5	
subitem not at all important	(●)	( )	( )	( )	( )	essential

Clear selection

Does your paper address subitem 19-i?

Copy and paste relevant sections from the manuscript (include quotes in quotation marks "like this" to indicate direct quotes from your manuscript), or elaborate on this item by providing additional information not in the ms, or briefly explain why the item is not applicable/relevant for your study

NA

19-ii) Include qualitative feedback from participants or observations from staff/researchers

Include qualitative feedback from participants or observations from staff/researchers, if available, on strengths and shortcomings of the application, especially if they point to unintended/unexpected effects or uses. This includes (if available) reasons for why people did or did not use the application as intended by the developers.

	1	2	3	4	5	
subitem not at all important	○	○	○	○	◉	essential

Clear selection

Does your paper address subitem 19-ii?

Copy and paste relevant sections from the manuscript (include quotes in quotation marks "like this" to indicate direct quotes from your manuscript), or elaborate on this item by providing additional information not in the ms, or briefly explain why the item is not applicable/relevant for your study

Twenty-eight (88%) of participants in the Slip Buddy condition attended post-intervention focus groups and they made 35 responses to the question about what they liked most about Slip Buddy App (IRR=94%; Kappa=.89). The most common theme of responses was ease of use/simple concept (n=23; 66%), followed by increasing accountability and/or awareness of overeating and/or triggers (n=8; 23%), the end-of-day reminder to track slips (n=2; 5%), feeling motivated to not slip so there would be nothing to track (n=1; 3%) and other (n=1; 3%). Participants made 35 responses about what they liked least about Slip Buddy App (IRR=91%; Kappa=.89). Responses reflected the following themes: technical issues (e.g., app crashing, notifications not going away; n=10; 29%), easy to forget to use or not sure how to use when no slips (n=7; 21%), did not find stress ratings relevant (n=5; 15%), focus on slips is too negative (n=4; 12%), was not sure what to count as a slip (n=4; 12%), did not include diet instruction (n=4; 12%), slip history screen was not as informative as it could be (n=1; 3%). For the final question regarding their thoughts on a feature that would allow them to track when they were tempted but did not slip, 75% (n=21) said they would be enthusiastic about this feature. The remainder said they worried it would add too much burden.

DISCUSSION

22) Interpretation consistent with results, balancing benefits and harms, and considering other relevant evidence

NPT: In addition, take into account the choice of the comparator, lack of or partial blinding, and unequal expertise of care providers or centers in each group

22-i) Restate study questions and summarize the answers suggested by the data, starting with primary outcomes and process outcomes (use)

Restate study questions and summarize the answers suggested by the data, starting with primary outcomes and process outcomes (use).

	1	2	3	4	5	
subitem not at all important	○	○	○	○	◉	essential

Clear selection

Does your paper address subitem 22-i? *

Copy and paste relevant sections from the manuscript (include quotes in quotation marks "like this" to indicate direct quotes from your manuscript), or elaborate on this item by providing additional information not in the ms, or briefly explain why the item is not applicable/relevant for your study

Findings revealed that while participants in both treatment conditions used their assigned apps on a similar percentage of intervention days (i.e., 54% of days for participants in the Slip Buddy condition and 58% of days for participants in the Calorie Tracking condition), 55% (n=18) of Slip Buddy participants used the app in week 12 of the intervention compared to only 35% (n=11) of Calorie Tracking participants, although this was not statistically significant. While less than one-third of Slip Buddy participants agreed that using the Slip Buddy App was tedious (29%, n=9), taxing (16%, n=5), or time-consuming (20%, n=6) and 77% (n=24) agreed tracking slips was easy, only 39% (n=12) agreed that tracking their slips with the app was helpful, which was significantly lower than the calorie tracking app condition. Slip Buddy also received lower usability ratings than a commercial calorie tracking app, perhaps not surprisingly, as commercial apps are years ahead of Slip Buddy in user experience optimization. Slip Buddy participants reported barriers such as technical difficulties, forgetting to use the app if they hadn't slipped in a while, and finding the exclusive focus on slips to be too negative. Slip data revealed that the majority of slips reported happened at home followed by work, and that snacks and dinner time were the eating episodes at which slips were most likely to occur. Activities that co-occurred with slips were distributed fairly evenly across work, socializing, screen time, and domestic tasks. Just under half of slips occurred under conditions of moderate to high stress and over half occurred while hungry.

22-ii) Highlight unanswered new questions, suggest future research

Highlight unanswered new questions, suggest future research.

	1	2	3	4	5	
subitem not at all important	○	○	○	○	◉	essential

Clear selection

Does your paper address subitem 22-ii?

Copy and paste relevant sections from the manuscript (include quotes in quotation marks "like this" to indicate direct quotes from your manuscript), or elaborate on this item by providing additional information not in the ms, or briefly explain why the item is not applicable/relevant for your study

Findings can also inform future studies on how technology can be leveraged to execute simpler forms of dietary self-monitoring given the high burden associated with traditional calorie tracking apps.

20) Trial limitations, addressing sources of potential bias, imprecision, and, if relevant, multiplicity of analyses

20-i) Typical limitations in ehealth trials

Typical limitations in ehealth trials: Participants in ehealth trials are rarely blinded. Ehealth trials often look at a multiplicity of outcomes, increasing risk for a Type I error. Discuss biases due to non-use of the intervention/usability issues, biases through informed consent procedures, unexpected events.

	1	2	3	4	5	
subitem not at all important	○	○	○	○	◉	essential

Clear selection

Does your paper address subitem 20-i? *

Copy and paste relevant sections from the manuscript (include quotes in quotation marks "like this" to indicate direct quotes from your manuscript), or elaborate on this item by providing additional information not in the ms, or briefly explain why the item is not applicable/relevant for your study

This study has limitations. First, the sample size was not large enough to compare the two conditions on weight loss. However, the purpose of this work was to evaluate feasibility and acceptability of the Slip Buddy App using a user-informed process to guide improvements to the technology prior to conducting a fully powered randomized trial. We chose a 12-week intervention length to allow us to gain user insights after a prolonged period of use, but this study does not provide information on tracking habits over longer periods of time, such as whether slip tracking decreases over time as participants learn their triggers and slip less. Another limitation is that our ability to measure app use via backend data was hampered by the fact that some participants did not see or receive end-of-the-day notifications that when clicked would indicate whether no slip entries meant a slip-free day or non-use. For this reason, we had to rely on self-report use data which is prone to biases due to forgetting or social desirability. This can be ameliorated in the next version, and the addition of temptation and exercise slip tracking will give the user more to do each day with the app even in the absence of slips. Another limitation is that the Slip Buddy app was not operating for two days which could have affected use in subsequent days to the extent that participants were frustrated by this. Because it is a newly developed app, bugs and crashes are more common relative to commercial apps that have been around for many years and this will certainly impact usability ratings. Finally, a limitation is that the sample overrepresented non-Hispanic White women (83% of our sample, n=53). In future research, recruitment will need to limit enrollment of non-Hispanic white women, the population segment that too often comprises the majority of weight loss trial samples,[37] to no more than the proportion of the population they represent.

21) Generalisability (external validity, applicability) of the trial findings

NPT: External validity of the trial findings according to the intervention, comparators, patients, and care providers or centers involved in the trial

21-i) Generalizability to other populations

Generalizability to other populations: In particular, discuss generalizability to a general Internet population, outside of a RCT setting, and general patient population, including applicability of the study results for other organizations

	1	2	3	4	5	
subitem not at all important	○	○	○	○	◉	essential

Clear selection

Does your paper address subitem 21-i?

Copy and paste relevant sections from the manuscript (include quotes in quotation marks "like this" to indicate direct quotes from your manuscript), or elaborate on this item by providing additional information not in the ms, or briefly explain why the item is not applicable/relevant for your study

Finally, a limitation is that the sample overrepresented non-Hispanic White women (83% of our sample, n=53). In future research, recruitment will need to limit enrollment of non-Hispanic white women, the population segment that too often comprises the majority of weight loss trial samples,[37] to no more than the proportion of the population they represent.

21-ii) Discuss if there were elements in the RCT that would be different in a routine application setting

Discuss if there were elements in the RCT that would be different in a routine application setting (e.g., prompts/reminders, more human involvement, training sessions or other co-interventions) and what impact the omission of these elements could have on use, adoption, or outcomes if the intervention is applied outside of a RCT setting.

	1	2	3	4	5	
subitem not at all important	○	○	○	○	◉	essential

Clear selection

Does your paper address subitem 21-ii?

Copy and paste relevant sections from the manuscript (include quotes in quotation marks "like this" to indicate direct quotes from your manuscript), or elaborate on this item by providing additional information not in the ms, or briefly explain why the item is not applicable/relevant for your study

NA

OTHER INFORMATION

23) Registration number and name of trial registry

Does your paper address CONSORT subitem 23? *

Copy and paste relevant sections from the manuscript (include quotes in quotation marks "like this" to indicate direct quotes from your manuscript), or elaborate on this item by providing additional information not in the ms, or briefly explain why the item is not applicable/relevant for your study

Trials Registration: Clinicaltrials.gov NCT02615171

24) Where the full trial protocol can be accessed, if available

Does your paper address CONSORT subitem 24? *

Cite a Multimedia Appendix, other reference, or copy and paste relevant sections from the manuscript (include quotes in quotation marks "like this" to indicate direct quotes from your manuscript), or elaborate on this item by providing additional information not in the ms, or briefly explain why the item is not applicable/relevant for your study

Trials Registration: Clinicaltrials.gov NCT02615171

25) Sources of funding and other support (such as supply of drugs), role of funders

Does your paper address CONSORT subitem 25? *

Copy and paste relevant sections from the manuscript (include quotes in quotation marks "like this" to indicate direct quotes from your manuscript), or elaborate on this item by providing additional information not in the ms, or briefly explain why the item is not applicable/relevant for your study

This work was funded by NIH grants R01HL122302 and K24HL124366 to Dr. Pagoto.

X27) Conflicts of Interest (not a CONSORT item)

X27-i) State the relation of the study team towards the system being evaluated

In addition to the usual declaration of interests (financial or otherwise), also state the relation of the study team towards the system being evaluated, i.e., state if the authors/evaluators are distinct from or identical with the developers/sponsors of the intervention.

	1	2	3	4	5	
subitem not at all important	○	○	○	○	◉	essential

Clear selection

Does your paper address subitem X27-i?

Copy and paste relevant sections from the manuscript (include quotes in quotation marks "like this" to indicate direct quotes from your manuscript), or elaborate on this item by providing additional information not in the ms, or briefly explain why the item is not applicable/relevant for your study

Dr. Pagoto is a paid scientific advisor for Fitbit and has been a paid consultant for WW (formerly Weight Watchers).

About the CONSORT EHEALTH checklist

As a result of using this checklist, did you make changes in your manuscript? *

- ○ yes, major changes
- ◉ yes, minor changes
- ○ no

What were the most important changes you made as a result of using this checklist?

Your answer

How much time did you spend on going through the checklist INCLUDING making changes in your manuscript *

90 minutes

As a result of using this checklist, do you think your manuscript has improved? *

- ○ yes
- ◉ no
- ○ Other:

Would you like to become involved in the CONSORT EHEALTH group?

This would involve for example becoming involved in participating in a workshop and writing an "Explanation and Elaboration" document

- ○ yes
- ◉ no
- ○ Other:

Clear selection

Any other comments or questions on CONSORT EHEALTH

cutting and pasting the relevant sections was a tedious task. seems a checklist would have sufficed.

STOP - Save this form as PDF before you click submit

To generate a record that you filled in this form, we recommend to generate a PDF of this page (on a Mac, simply select "print" and then select "print as PDF") before you submit it.

When you submit your (revised) paper to JMIR, please upload the PDF as supplementary file.

Don't worry if some text in the textboxes is cut off, as we still have the complete information in our database. Thank you!

Final step: Click submit !

Click submit so we have your answers in our database!

Submit

Never submit passwords through Google Forms.

This content is neither created nor endorsed by Google. Report Abuse - Terms of Service - Privacy Policy

Google Forms
